# Role of Occult and Post-acute Phase Replication in Protective Immunity Induced with a Novel Live Attenuated SIV Vaccine

**DOI:** 10.1371/journal.ppat.1006083

**Published:** 2016-12-21

**Authors:** Neil Berry, Maria Manoussaka, Claire Ham, Deborah Ferguson, Hannah Tudor, Giada Mattiuzzo, Bep Klaver, Mark Page, Richard Stebbings, Atze T. Das, Ben Berkhout, Neil Almond, Martin P. Cranage

**Affiliations:** 1 Division of Virology, National Institute for Biological Standards and Control, South Mimms, United Kingdom; 2 Institute for Infection & Immunity, St George’s, University of London, London, United Kingdom; 3 Laboratory of Experimental Virology, Department of Medical Microbiology, Center for Infection and Immunity Amsterdam (CINIMA), Academic Medical Center, University of Amsterdam, Amsterdam, The Netherlands; 4 Division of Biotherapeutics, National Institute for Biological Standards and Control, South Mimms, United Kingdom; Miller School of Medicine, UNITED STATES

## Abstract

In order to evaluate the role of persisting virus replication during occult phase immunisation in the live attenuated SIV vaccine model, a novel SIVmac239Δ*nef* variant (SIVrtTA) genetically engineered to replicate in the presence of doxycycline was evaluated for its ability to protect against wild-type SIVmac239. Indian rhesus macaques were vaccinated either with SIVrtTA or with SIVmac239Δ*nef*. Doxycycline was withdrawn from 4 of 8 SIVrtTA vaccinates before challenge with wild-type virus. Unvaccinated challenge controls exhibited ~10^7^ peak plasma viral RNA copies/ml persisting beyond the acute phase. Six vaccinates, four SIVmac239Δ*nef* and two SIVrtTA vaccinates exhibited complete protection, defined by lack of wild-type viraemia post-challenge and virus-specific PCR analysis of tissues recovered *post-mortem*, whereas six SIVrtTA vaccinates were protected from high levels of viraemia. Critically, the complete protection in two SIVrtTA vaccinates was associated with enhanced SIVrtTA replication in the immediate post-acute vaccination period but was independent of doxycycline status at the time of challenge. Mutations were identified in the LTR promoter region and *rtTA* gene that do not affect doxycycline-control but were associated with enhanced post-acute phase replication in protected vaccinates. High frequencies of total circulating CD8^+^T effector memory cells and a higher total frequency of SIV-specific CD8^+^ mono and polyfunctional T cells on the day of wild-type challenge were associated with complete protection but these parameters were not predictive of outcome when assessed 130 days after challenge. Moreover, challenge virus-specific Nef CD8^+^ polyfunctional T cell responses and antigen were detected in tissues *post mortem* in completely-protected macaques indicating post-challenge control of infection. Within the parameters of the study design, on-going occult-phase replication may not be absolutely required for protective immunity.

## Author Summary

Development of an HIV vaccine remains a global health priority. In non-human primates live-attenuated SIV induces a potent vaccine effect. Following disappearance of vaccine virus from the peripheral circulation replication persists in lymphoid tissue. To address whether this occult replication is critical to the generation of protective immunity we used a novel construct (SIVrtTA) based on the prototypic live attenuated SIVmac239Δ*nef* but which requires the presence of the antibiotic doxycycline to replicate. Protection appeared independent of doxycycline status at the time of virulent virus challenge suggesting that occult replication may not be absolutely necessary for persistence of immunity; however, stronger protection was observed in monkeys vaccinated with SIVrtTA where vaccine replication persisted for longer after peak viraemia. Moreover, some evidence of very low level breakthrough of vaccine virus replication was seen and protection was weaker than that obtained with SIVmac239Δ*nef*. Both vaccination and challenge perturbed circulating T cell populations, but only the frequency of SIV-specific CD8^+^ polyfunctional T cells measured on the day of challenge was associated with protection. Replication-conditional mutants such as SIVrtTA have great potential in unlocking the complex interactions between the vaccine virus and host responses in the generation of potent anti-viral protection *in vivo*.

## Introduction

Live attenuated SIV has proven to be a highly effective vaccination strategy in non-human primate (NHP) models of HIV/AIDS [1,2], in many cases protecting macaques from detectable superinfection following re-challenge with both homologous and heterologous wild-type SIV administered systemically and mucosally [3–23]. Although safety concerns such as reversion to virulence and recombination with wild-type strains preclude direct application of this vaccine approach in humans, a clearer understanding of mechanisms of pathogenesis and protection may inform the development of more clinically acceptable HIV vaccines. Studies have been performed using vaccine viruses attenuated by genetic disruption of key regulatory genes including *nef*, *vpx*, *vpr and vif*; although the moderately attenuated prototypic vaccine strain SIVmac239Δ*nef* has been used for the majority of studies.

Attempts to establish clearly defined immune correlates of protection have not been conclusive, particularly where studies have measured responses in peripheral blood. Indeed, the only robust correlate identified so far is the observation between increasing attenuation of the vaccine virus and decreasing protection [11]. Recently, a detailed comparative study of different attenuated virus strains derived from SIVmac239 concluded that protection was associated with the induction of an effector memory T cell (T_EM_) response and protection of the T follicular helper (T_FH_) cell subset in lymphoid tissue [10]. This association, however, is not definitively established as the mechanism of protection.

A crucial property of minimally-attenuated SIV vaccines, which are the most effective, is the widespread distribution of the vaccine virus in multiple lymphoid tissues [22] but the role of occult replication (*i*.*e*. replication in lymphoid tissue when virus is no longer or only intermittently detected in the peripheral circulation) in the generation of protective immunity is not fully understood. Vaccine virus persistence may result in multiple alterations in the host innate immune system that contribute to protection, in addition to the induction of adaptive immune responses [22]. In the study reported here we have sought to influence occult phase persisting turnover of live attenuated SIV using a novel approach: the conditionally live attenuated SIVmac239Δ*nef* vaccine (SIVrtTA) that *in vitro* is absolutely dependent on the presence of doxycycline (dox) to replicate [24, 25]. Previously, we have shown that SIVrtTA is infectious in Indian rhesus macaques and induced reversible up-regulation of the frequency of global circulating T_EM_ [26].

Here, we report the outcome of an intravenous challenge of two groups of SIVrtTA-vaccinated macaques with wild-type SIVmac239 in comparison with macaques vaccinated with the prototypic SIVmac239Δ*nef* live attenuated vaccine. One group of SIVrtTA vaccinates macaques remained on daily administration of dox, whereas another group received the final dose of dox 8 weeks prior to wild-type virus challenge during the occult phase of virus replication. Protection against detectable infection with wild-type, highly virulent SIVmac239 was observed at various levels; however, the pattern of protection did not associate directly with the experimental treatment protocol, but with the kinetics of vaccine-virus replication in the acute and immediate post-acute period of vaccine viraemia and with vaccine-driven T cell immune responses.

## Results

### Viral vaccine kinetics

**Fig 1:**
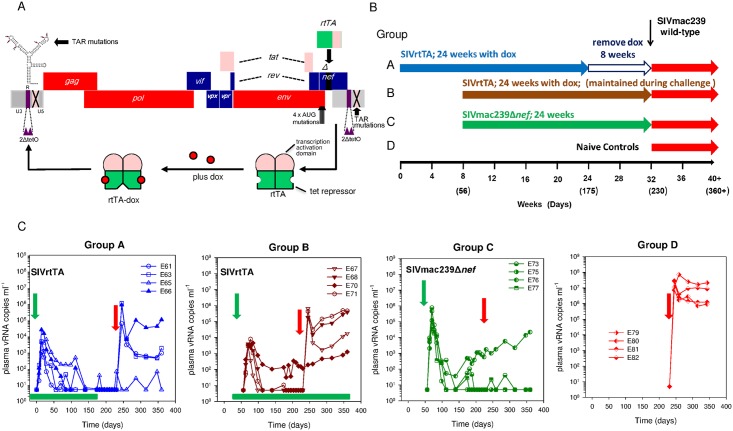
SIVrtTA organisation, study design and group vRNA profiles. (A). Diagrammatical representation of the SIVrtTA genome genetically engineered from a SIVmac239 backbone. The Tat-TAR transcriptional mechanism was inactivated by multiple mutations in TAR. The *rtTA* gene was introduced at the site of the *nef* gene and tet-operator (tetO) elements were placed in the U3 promoter region. Upon binding of doxycycline (dox), the rtTA protein undergoes a conformational switch that allows the protein to bind to the tetO elements and subsequently activate gene expression and virus replication. (B). Schematic outline of the study plan, with two groups of SIVrtTA vaccinates (Groups A and B) with 24 weeks (six months) exposure to dox, removed for 8 weeks in Group A (clear arrow) but maintained in Group B. Group C were vaccinated withSIVmac239Δ*nef* for 24 weeks (6 months). Group D represents naïve controls challenged with wild-type SIVmac239 for a further 20 weeks. Time-scale is shown in weeks with the key time-point indicated as days in parentheses. (C). Plasma vRNA profiles for SIVrtTA vaccine groups A and B, SIVmac239Δ*nef*, group C and naïve challenge controls (group D) respectively. Green arrows indicate time of vaccine administration. Periods of doxycycline administration are shown in green blocks (Groups A and B only) for 2 weeks prior to SIVrtTA vaccination up to day 175 in Group A and throughout both vaccine and challenge periods in Group B. Wild-type SIVmac239 (red arrows) was administered to Groups A, B and C 230 days after the start of vaccine-immunisation period of Group A (*ie* study start). Group D controls were challenged at the same time as all vaccinates.

Two groups (A & B) of four Indian-derived rhesus macaques were injected intravenously with 5 x 10^3^ TCID_50_ SIVrtTA vaccine (genetically engineered from the SIVmac239 backbone as indicated in Fig 1A) and treated with dox for 6 months followed by a period of 8 weeks without dox (Group A; E61, E63, E65, E66) or treated with dox for 6 months and then maintained on dox(Group B; E67, E68, E70, E71). A further 4 macaques (Group C; E73, E75, E76, E77) were vaccinated with SIVmac239Δ*nef* for 6 months and four unvaccinated, naïve macaques (E79-E82) were included as challenge controls (Fig 1B). Total SIV *gag* vRNA profiles are shown for Groups A-C as a continuum of vaccination and wild-type challenge profiles (Fig 1C). As previously reported [26], the SIVrtTA vaccinates displayed a transient peak in plasma vRNA kinetics which is characteristic for attenuated SIVmac239Δ*nef* with two exceptions: E65 (Group A) and E70 (Group B). These animals exhibited a persisting shoulder of ~ 10^2^ vRNA copies/ml to ~100 days post-vaccination. From day 110 post-infection (p.i), prior to removal of dox, E65 plasma vRNA fell below the limit of detection. Plasma vRNA remained stably elevated in E70, which was maintained on daily dox treatment to the time of wild-type challenge. Another macaque, vaccinated with SIVmac239Δ*nef* (E76, Group C) also failed to completely control viraemia below the limit of detection. Hence, at the time of wild-type SIVmac239 challenge detectable vRNA signals were present in the plasma of vaccinates E65, E70 (SIVrtTA) and E76 (SIVmac239Δ*nef*) (Fig 1, S1 Fig).

### Challenge outcome

Challenge outcome was initially assessed by the individual comparison of total SIV *gag* plasma vRNA profiles for each group (Fig 1C). As expected, all four naive, unvaccinated control macaques challenged with wild-type SIVmac239 (Group D) exhibited high plasma vRNA loads (1.86 x10^7^ mean SIV RNA copies/ml) by day 14 p.i. which also exhibited a high vRNA steady state (10^6^–10^8^ wtSIVmac239 copies/ml) throughout the 20 week follow-up period. In contrast, a marked vaccine effect was observed in all animals vaccinated with either SIVrtTA or SIVmac239Δ*nef* (Fig 1C, Groups A and B or C respectively).

Statistical analyses of suppression of vRNA levels post wild-type challenge were determined using a Kruskal-Wallis analysis with a Dunn’s post-hoc test to determine significance levels. Peak viraemia was statistically significantly suppressed when Groups A, B and C were compared individually to Group D, although the vaccination with SIVmac239Δ*nef* resulted in the most significant outcomes. P values for Groups A-C were, p = 0.05, p = 0.05 and p = 0.001 respectively at day 14. When viraemia levels were analysed at day 84 (steady-state) significance was retained in Groups A and C (p = 0.012 and p = 0.002 respectively) although, interestingly, significance was lost at day 84 in Group B (p = 0.135). All SIVrtTA vaccinates analysed together (A and B combined) exhibited significant differences from Group D challenge controls at peak (day 14) and steady-state (day 84) time-points (p = 0.025 and 0.021 respectively).

E73, E76 and E77 (vaccinated with SIVmac239Δ*nef*) and E65 (vaccinated with SIVrtTA) exhibited plasma vRNA levels that remained <100 SIV RNA copies/ml. Macaques E76 (vaccinated with SIVmac239Δ*nef*) and E70 (vaccinated with SIVrtTA) exhibited plasma viral loads of the vaccine virus higher than 100 SIV RNA copies/ml prior to wild-type SIV challenge, with vRNA viral loads gradually increasing in the 20 week follow-up period. Of the remaining macaques vaccinated with SIVrtTA, with undetectable plasma vRNA on the day of challenge (E61, E63, E66, E67, E68, E71), a significant peak in plasma viremia was detected 14 days after wild-type challenge (mean 5.43 x 10^5^ SIV RNA copies per ml), which was partially resolved, but remained between 1 x 10^3^–1 x 10^6^ SIV RNA copies/ml 20 weeks post-challenge.

**Fig 2:**
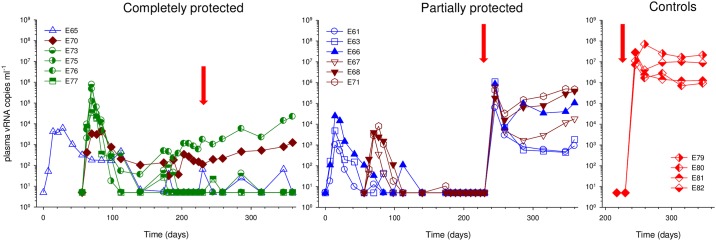
Plasma vRNA profiles in completely and partially protected macaques and challenge controls. Complete time-course of total SIV RNA *gag* levels determined by real-time RT-PCR detailing kinetics of the vaccine and challenge viruses (red arrows signify start of wild-type (WT) SIVmac239 challenge period) showing completely protected, partially protected and unvaccinated naïve control macaques. Administration of challenge virus is indicated by red arrows.

From these initial analyses it was possible to classify macaques into two levels of protection: (1) complete-protection defined as having no secondary peak after wild-type virus challenge (E65, E70: SIVrtTA; E73, E75, E76, E77: SIVmac239Δ*nef*). (2) partially-protected macaques that exhibited a clear secondary peak of viraemia 14 days post SIVmac239 wild-type challenge (E61, E63, E66, E67, E68, E71; SIVrtTA). When these data were re-plotted, also as a continuum, two patterns of plasma vRNA profiles were revealed immediately prior to and post wild-type SIVmac239 challenge, reflecting these two general classifications of protection as represented in Fig 2. Interestingly, in the partially protected group, the secondary spike in vRNA is immediately preceded by a virtual absence in detectable vaccine-virus replication prior to wild-type challenge. By comparison, in the completely protected group, total SIV *gag* vRNA signals are clearly evident in the same period (~100 days) up to challenge with little perturbations in these levels post-challenge. However, as total plasma SIV RNA levels reveal only part of the overall biomarker of infection picture, discriminatory PCR assays were required to fully evaluate the protection status of each macaque.

### Differential detection of viral DNA and RNA of vaccine and wt species

**Fig 3:**
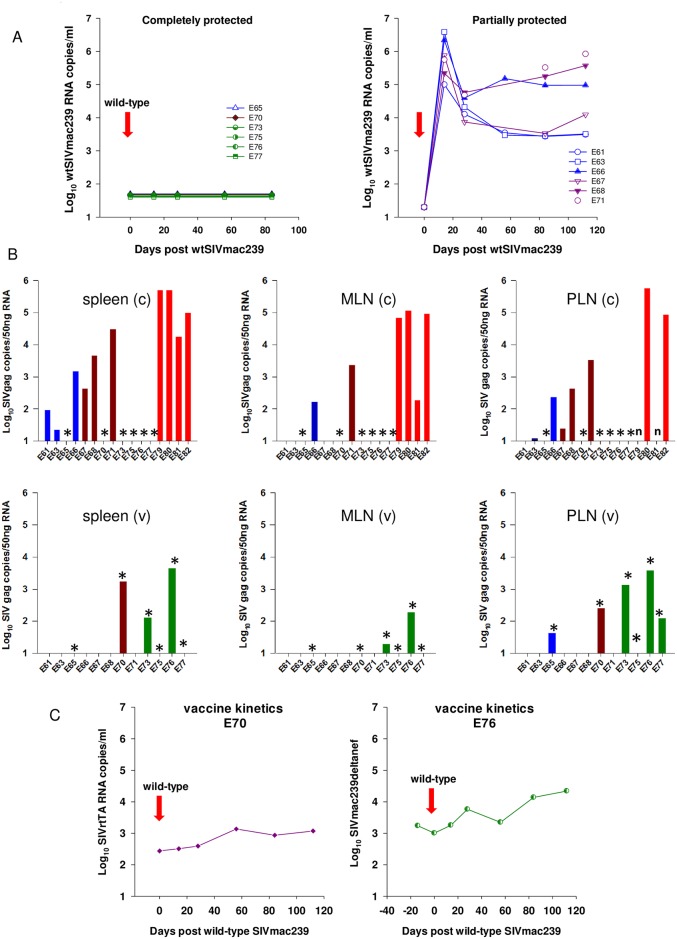
Virus–specific vRNA levels and vaccine sequestration. (A). Virus-specific plasma vRNA levels determined by real-time PCR in the period post wild-type SIVmac239 challenge (red arrows). Levels of wild-type-specific plasma viral RNA quantitatively assessed with SIVmac239-specific vRNA assays separated vaccinates into completely protected (*n = 6*; E65, E70, E73, E75, E76, E77) or partially protected (n = 6; E61, E63, E66, E67, E68, E71) groups. (B). Cell-associated RNA (CA-RNA) expressed as viral RNA copies/50ng RNA normalised to GAPDH for either challenge (c) or vaccine (v) viruses for spleen, mesenteric lymph nodes (MLN) and peripheral lymph nodes (PLN), represented by initial colour coded group (*ie* Group A, blue; Group B; dark red; Group C, green, Group D, bright red). Asterisks (*) identify completely protected macaques as represented in panel A. E75 (SIVmac239Δ*nef*) was qualitatively positive only in PLN, signalling at the limit of assay detection. n indicates no sample available for testing. (C). Vaccine virus replication profiles for SIVrtTA vaccinate E70 and SIVmac239Δ*nef* vaccinate E76 are shown, exhibiting sustained levels of vaccine replication post-wtSIVmac239 challenge (red arrows).

To discriminate further between superinfection with wild-type virus and recrudescence/persistence of vaccine virus, discriminatory PCR assays were established that selectively detect either vaccine-derived or wild-type vRNA in plasma, total vDNA signals in tissues or cell-associated viral RNA (CA-RNA). From these combined analyses a clear picture of superinfection status emerged with the ability to detect and quantify each viral nucleic acid species in blood and/or selected lymphoid tissues (Figs 2 and 3; S2 Fig). Wild-type SIVmac239-specific vRNA determinations partitioned macaques into completely protected (E65, E70, E73, E75, E76, E77) or partially protected (E61, E63, E66, E67, E68, E71) as indicated in Fig 3A. There was a highly statistically significant difference between completely protected macaques and naïve challenge controls (p<0.001 at days 14 and 84 post SIVmac239 wild-type challenge) using a Kruskal-Wallis analysis with a Dunn’s post-hoc test. Partially-protected macaques all demonstrated a spike in plasma vRNA that was unambiguously attributed to establishment of wild-type virus infection which at days 14 and 84 were non-significant (p = 0.095) compared to challenge controls, applying the same statistical test as for the completely protected group. Additionally, a broad range of lymphoid tissues was assessed for wild type SIV DNA (S2 Fig). High levels were detected in all tissues in naïve challenge controls, with lower but detectable levels in most tissues in E61, E63, E66, E67, E68 and E71, reflecting profiles of plasma viral RNA. No wild type SIV DNA was detected in any tissue from the completely-protected animal vaccinated with SIVrtTA (E65) and a single signal of wild-type SIVmac239 DNA detected in the spleen of E70. No wtSIVmac239-specific DNA was detected in animals of Group C vaccinated with SIVmac239Δ*nef*.

Additional information relating to the ability to detect apparently replication-competent virus, rather than proviral signals, was gained for a number of tissues by measuring CA-RNA concentrations for vaccine and wild-type viruses 20 weeks after SIVmac239 challenge (Fig 3B). Wild-type SIV was never detected by any molecular biomarker of infection in those macaques vaccinated with SIVmac239Δ*nef* (E73, E75, E76, E77), further confirming the complete protection status of this group. No wtSIVmac239 CA-RNA was detected in macaques E65 and E70 vaccinated with SIVrtTA, compared with high levels of wtSIVmac239 detected in all naïve challenge controls, particularly in the spleen and mesenteric lymph nodes (MLN). Lower levels of wtSIVmac239 CA-RNA were detected in spleen samples from E61, E63, E66, E67, E68 and E71 and more sporadically from MLN and peripheral lymph node (PLN) samples (Fig 3B). Hence, with information gained from virus-specific differential PCR techniques, taking only wild-type SIVmac239 levels as measures of outcome, there was a statistically significant difference in outcome between completely protected macaques and wild-type challenge controls and partially protected and wild-type challenge controls.

### Evidence for SIVrtTA replication in protected vaccinates

Although E65 and E70 displayed undetectable signals for wild-type specific plasma and CA-RNA, both vaccinates signalled positive by SIVrtTA-specific RT-PCR, particularly E70 in the plasma, spleen and PLN (Fig 3B and 3C). These data reflect the plasma vRNA signal in E70 post-wtSIV239 challenge which was unambiguously attributable to continuous SIVrtTA replication in the continued presence of dox. Remarkably, SIVrtTA replication did not fluctuate over time in this macaque, nor was it perturbed by administration of the wild-type challenge virus (Figs 1C, 2 and 3). In this respect, E70 was comparable to macaque E76 (Group C; SIVmac239Δ*nef*) that displayed similar continuous viral kinetics post SIVmac239 challenge despite resistance to wild-type superinfection as confirmed by lack of wild-type SIVmac239 RNA signals in either plasma or tissues.

Perhaps the most interesting vaccinate of all groups was SIVrtTA-vaccinated macaque E65, which resisted wtSIVmac239 but displayed highly controlled vRNA kinetics in the later post-acute phase. However, in the absence of dox, four blips of plasma vRNA were noted as determined by total SIV-*gag* qPCR (Figs 1C and 2), two prior to wild-type challenge but after dox removal and two blips after wild-type challenge. Analysis of tissues for CA-RNA indicated low, but clearly detectable SIVrtTA in the PLN at termination. Taken together, these data suggest evidence of very low, but persistent replication of SIVrtTA in E65 when there was no, or little, dox present. Moreover, both SIVrtTA vaccinates E65 and E70 had detectable levels of SIVrtTA-specific CA-RNA at termination, many weeks after initial vaccine administration. Extending these observations to Group C vaccinates (SIVmac239Δ*nef*) all had some level of residual detectable vaccine virus replication at termination (Fig 3). Indeed, all 6 completely protected vaccinates signalled positive for the vaccine virus *post-mortem* in PLN suggesting this to be an important site for virus sequestration, which as well as the spleen represents an important reservoir for the vaccine virus.

### Protected vaccinates did not boost anti-p27 responses

All vaccinates seroconverted to SIV *Gag* p27 prior to challenge with wild-type SIV (S3 Fig). Anti-p27 responses were broadly similar amongst all macaques vaccinated with SIVrtTA regardless of dox withdrawal and anti-p27 titres were lower than those in SIVmac239Δ*nef* vaccinates. All fully protected animals, E65 and E70 vaccinated with SIVrtTA and E73, E75, E76 and E77 vaccinated with SIVmac239Δ*nef*, showed only minor perturbations in anti-p27 titre after challenge with wild-type SIVmac239, whereas a marked increase in anti-p27 titres was detected in all other macaques (S3 Fig).

### Sequence analysis of SIVrtTA upon *in vivo* replication

In order to address the possibility that mutations arising in SIVrtTA as a result of selection *in vivo* may have occurred, SIVrtTA RNA recovered from vaccinates was sequenced. For this, plasma vRNA was isolated at several times during the immediate post-acute phase period, when qRT-PCR revealed a vRNA load of >10^2^ SIV RNA copies/ml including where there was the persisting shoulder of prolonged SIVrtTA replication in E65 and E70 SIVrtTA vaccinates.

In SIVrtTA, the Tat-TAR transcription activation mechanism has been functionally replaced by the dox-inducible Tet-On gene expression system [24, 25, 27]. To achieve this (1) TAR was inactivated through mutations in the binding sites for Tat and pTEFb, (2) the gene encoding the dox-inducible rtTA transcriptional activator was inserted at the site of the accessory *nef* gene and (3) tet operator (tetO) elements to which the dox-rtTA complex can bind were inserted between the NFκB and Sp1 binding sites in the U3 domain of the LTR promoter (Fig 1A). Sequencing of the LTR and leader RNA region of different SIVrtTA RNA samples demonstrated the stable presence of the TAR-inactivating mutations and no additional changes were observed in TAR. The virus also stably maintained the tetO elements but whereas the vaccine strain contained a triplicated NFκB-tetO repeat (resulting from *in vitro* evolution; [28]), deletion of one of these repeats was frequently observed (S1 Table; S4 Fig). Previous experiments demonstrated that such a deletion slightly reduces the transcriptional activity of the LTR promoter, but does not affect dox-control. In all macaques, a point mutation was observed in the primer binding site (PBS) sequence (T731C). This mutation was due to the fact that the SIVrtTA vaccine construct contained a PBS complementary to the infrequently used tRNAlys5 primer for reverse transcription [29, 30]. As expected, the *in vivo* replicating virus demonstrated a PBS sequence corresponding to the more frequently used tRNAlys3 primer. Sequencing of the *tat* gene did not reveal any sequence changes.

However, sequence analysis of the rtTA gene identified two non-silent codon changes (R80W and E191K) in the E65 samples isolated at 6 weeks after vaccination and later (S1 Table). The E70 sample isolated at 6 weeks after vaccination demonstrated an R80Q change, whereas later E70 samples (from 14 weeks) also demonstrated the R80W substitution. We did not identify such rtTA mutations in the other macaques vaccinated with SIVrtTA. The identified amino acid changes had never been observed previously in multiple long-term *in vitro* evolution experiments with SIVrtTA or with a similar dox-controlled HIVrtTA variant and hence represents a novel finding.

Testing the transcriptional activity of the new R80W and E191K rtTA variants demonstrated that the mutations did not increase the background activity in the absence of dox (no loss of dox control) nor significantly alter the dox-induced activity (S5 Fig). As both E65 and E70 showed prolonged SIVrtTA replication, the mutations may improve *in vivo* replication of the virus. Importantly, these results demonstrate that the *in vivo* replicating virus stably maintains the integrated dox-control mechanism and did not restore the Tat-TAR axis of transcription control.

### No association between outcome and TRIM5 status

Since TRIM5α status and MHC type may influence vaccine challenge outcome [31, 32], the TRIM5α/TRIMcyp status and MHC type of all study macaques was determined (S2 Table). While no direct associations were identified between either MHC or TRIM5/cyp status and outcome it is interesting to note that the two macaques which failed to control the vaccine virus (E70: SIVrtTA; E76: SIVmac239Δ*nef*) and were protected from wild-type SIVmac239 did not express any of the major *mamu* A alleles analysed (S1 Fig). In this study, we could not identify any confounding factors associated with TRIM5α or TRIMcyp genotype.

### High frequency of total circulating CD8^+^ T_EM_ on day of wt challenge was associated with, but alone not predictive of, complete protection

**Fig 4:**
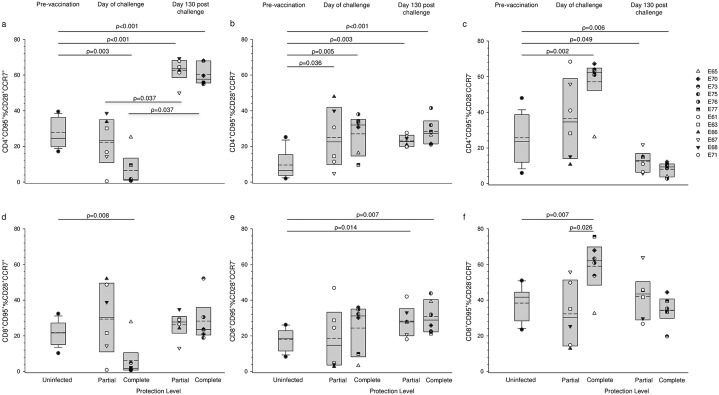
Analysis of global peripheral blood memory T cell populations with respect to protection status following superinfection challenge. Memory subsets were defined on differential expression of CCR7 and CD28 within the CD95^+^ T cell population. Results are shown for individual animals on the day of superinfection challenge and at day 130, after challenge and as median and mean values with boxes showing 25^th^ and 75^th^ percentiles grouped according to outcome of challenge. Pre-vaccination frequencies are shown as box plots derived from 18 macaques entering the original study. a, b, & c show frequencies of T_CM_, T_EM1_ and T_EM2_ subsets of CD4^+^ cells respectively; likewise d, e, & f show frequencies of T_CM_, CD28^+^CCR7^-^, and T_EM_ subsets of CD8^+^ cells respectively. All results were compared and statistically significant differences for groups are shown as ρ values determined by Mann-Whitney rank sum test.

We have previously reported that under replication permissive conditions, during the period when live attenuated virus RNA was essentially below the limit of detection in plasma, the global circulating T effector memory (T_EM_) cell frequency was upregulated [26]. Hence, we were interested to determine if this effect was associated with the degree of protection from superinfection. Comparison of partially and completely protected macaques on the day of challenge revealed that for both CD4^+^ and CD8^+^ CD95^+^ T cells, completely protected macaques had a lower median frequency of T_CM_ and reciprocally a higher median frequency of CD28^-^ CCR7^-^ T_EM_ than partially protected macaques; however, the difference in T_EM_ frequencies between these groups only reached significance in CD8^+^ T cells (ρ = 0.026; Mann-Whitney rank sum test) (Fig 4). Comparison with results from naïve macaques showed that median frequencies of both CD4^+^ and CD8^+^ T_CM_ were significantly reduced in completely protected macaques (ρ = 0.003 and ρ = 0.008 respectively; Mann-Whitney rank sum test). Conversely, the frequencies of CD4^+^ and CD8^+^ T_EM_ (CD28^-^ CCR7^-^) were significantly elevated in completely protected macaques compared with naïve macaques (ρ = 0.002 and ρ = 0.007; Mann-Whitney rank sum test). Despite these differences at the population level exceptions were seen: T cell frequencies for macaque E65, challenged under conditions of dox withdrawal were similar to those for naïve or partially protected macaques. Conversely, partially protected macaque E67 challenged under dox maintenance had a high frequency of CD28^-^ CCR7^-^ CD8^+^ T_EM_ and partially protected macaque E71 also challenged under replication permissive conditions had relatively high frequencies of both CD28^-^ and CD28^+^, CCR7^-^ CD95^+^ CD8^+^ T cells. So, although there was an association between a high frequency of global CD8^+^ T_EM_ in the circulation on the day of challenge and complete protection, a high T_EM_ frequency alone was not predictive of complete protection status.

### Perturbation of circulating global memory T cell populations following challenge regardless of protection status

The phenotype of circulating T cells was examined again at day 130 following superinfection challenge. At this time-point no significant differences were found between partially and completely protected macaques; moreover, global circulating CD4^+^ memory T cell populations were significantly perturbed (Fig 4). CD4^+^ T_CM_ were significantly elevated in superinfection-challenged animals, regardless of protection status compared with frequencies in naïve macaques (ρ = <0.001; Mann-Whitney rank sum test). Likewise, comparison of T_CM_ frequencies on the day of challenge with day 130 post-challenge showed significantly elevated frequencies regardless of protection status (ρ = <0.031; Wilcoxon signed rank test). The median frequencies of CD4^+^ CD28^+^CCR7^-^ (intermediate) T cells remained elevated post challenge compared with naïve animals (ρ = 0.003 and ρ = <0.001 for partially and completely protected groups respectively; Mann-Whitney rank sum test) and showed no statistical difference between day of challenge and day 130 post challenge for either group. In contrast, the median frequencies of T_EM_ were significantly reduced 130 days after challenge compared with those in naïve animals; although this was most marked in completely protected animals (ρ = 0.049 and ρ = 0.006, partially and complete protection groups respectively; Mann-Whitney rank sum test). Similarly, pairwise comparison of completely protected animals revealed a significant reduction in T_EM_ cell proportions between the day of challenge and 130 days post challenge (ρ = 0.031; Wilcoxon signed rank test). Five of 6 partially protected macaques also had lower frequencies at day 130 post challenge (ρ = 0.063; Wilcoxon signed rank test).

A somewhat different pattern of perturbation in circulating CD8^+^ T memory cell populations was seen following superinfection challenge (Fig 4). These changes were again, as for CD4^+^ T cells, independent of superinfection status. Frequencies of T_CM_ and T_EM_ (CD28^-^CCR7^-^) were not significantly different from frequencies in naïve animals; whereas CD28^+^CCR7^-^ cell frequencies were significantly elevated compared with naïve macaques for both partially and completely protected macaques (ρ = 0.014 and ρ = 0.007 respectively; Mann-Whitney rank sum test) and were not significantly different from day of challenge frequencies. Five of 6 completely protected macaques, the exception being macaque E65, had elevated T_CM_ and reduced T_EM_ (CD28^-^CCR7^-^) at day 130 post-challenge compared with day of challenge but failed to reach statistical significance (p = 0.063 Wilcoxon signed rank sum test). Pairwise comparison of T_CM_ and T_EM_ frequencies at day 130 post-challenge and day of challenge in partially protected macaques showed no significant changes. Thus, the polarisation of circulating CD8^+^ T memory populations observed in completely protected macaques on the day of challenge was not evident 130 days after superinfection challenge.

### Completely protected macaques had a higher total frequency of circulating SIV-specific CD8^+^ mono and polyfunctional T cells on the day of challenge compared with partially protected macaques

**Fig 5:**
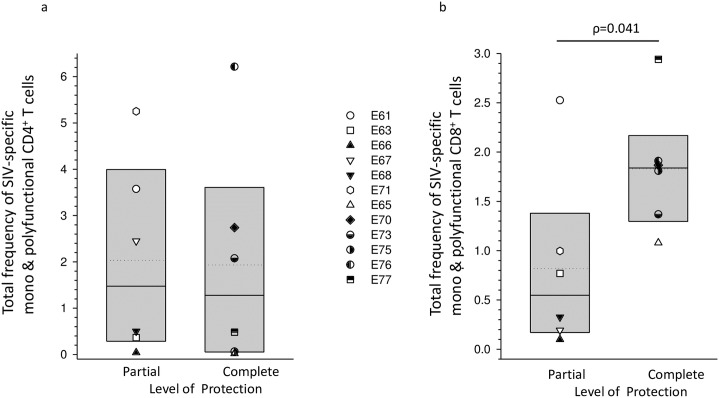
Analysis of total frequencies of SIV-specific mono and polyfunctional circulating T cells on the day of superinfection challenge. Results for CD4^+^ cells (a) and for CD8^+^ cells (b) are shown for individual macaques and as groups based on superinfection protection status. Total frequencies were derived by addition of mono, bi, tri and quadruple functional cells for each peptide pool tested. Box plots show median and mean values with 25^th^ and 75^th^ percentiles. Statistically significant differences for groups are shown as ρ values determined by Mann-Whitney rank sum test.

In order to evaluate the possible influence of SIV-specific T cell quantity and quality on protection status, PBMC were stimulated *in vitro* with peptide pools from SIV Gag, Rev and Tat and intracellular cytokine staining for IL-2, IFN-γ, TNF-α and IL-17 was analysed by flow cytometry for CD4^+^ and CD8^+^ T cells. The total frequency (*ie* mono + bi + tri + quadruple) of SIV-specific CD8^+^ T cells was found to be significantly higher in completely protected compared with partially protected macaques on the day of challenge (ρ = 0.041; Mann-Whitney rank sum test); whereas no difference was seen with CD4^+^ cells (Fig 5). A similar analysis at day 130 after challenge failed to show a difference between groups for either CD8^+^ or CD4^+^ T cells (S6 Fig). It was noted, however, that the frequency of CD4^+^ T cells was markedly elevated in both groups regardless of protection status when compared with day of challenge and was statistically significantly different for completely protected animals (p = 0.063 for partially protected and p = 0.031 for fully protected animals; Wilcoxon signed rank test). In only one animal, E61, were frequencies similar on the two occasions tested (3.57% and 3.51%, day of challenge and day 130 post challenge respectively) and were largely confined to mono-functionality (see below). Although total frequencies of SIV-specific CD8^+^ T cells also showed an upwards trend at day 130 after challenge the differences were not statistically significant.

### Quadruple cytokine positivity and IFN-γ + TNF-α dual positivity in circulating SIV-specific CD8^+^ T cells were associated with complete protection

**Fig 6:**
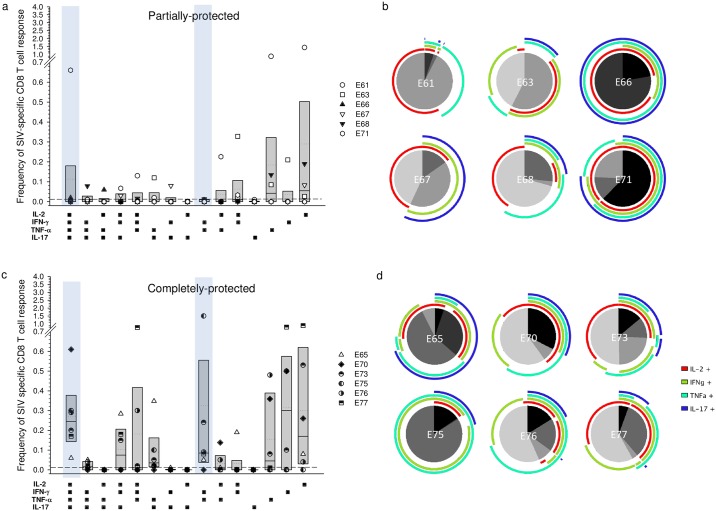
Frequency and distribution of SIV-specific CD8^+^ peripheral blood T cells on the day of superinfection challenge with respect to protection status. Cell populations were determined by multi-parametric flow cytometry following stimulation of PBMC separately with SIV-Gag, Rev and Tat peptide pools. Background responses detected in medium alone control samples were subtracted for every combination of cytokines and a cut-off of >0.01% after background subtraction was used as the threshold for positive reactivity (dashed line). Frequencies were derived by addition of results for Gag, Rev and Tat. Box plots show the 25^th^ and 75^th^ percentiles and median (solid line) and mean (dotted line) for each cytokine combination (a & c). Statistically significant differences are highlighted by blue boxes. Proportionate functionality for each macaque (b & d) is shown as a pie chart, with quadruple positivity shown in black and triple to mono positivity shown as shades of grey. Arcs show the combination of cytokine reactivities.

**Fig 7:**
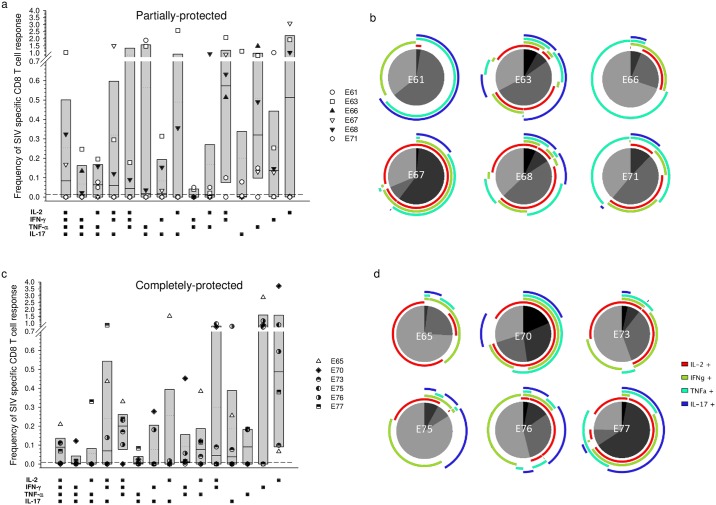
Frequency and distribution of SIV-specific CD8^+^ peripheral blood T cells 130 days after superinfection challenge with respect to protection status. Cell populations were determined by multiparametric flow cytometry following stimulation of PBMC separately with SIV-Gag, Rev and Tat peptide pools. Background responses detected in medium alone control samples were subtracted for every combination of cytokines and a cut-off of >0.01% after background subtraction was used as the threshold for positive reactivity (dashed line). Frequencies were derived by addition of results for Gag, Rev and Tat. Box plots show the 25^th^ and 75^th^ percentiles and median (solid line) and mean (dotted line) for each cytokine combination (a & c). Proportionate functionality for each macaque (b & d) is shown as a pie chart, with quadruple positivity shown in black and triple to mono positivity shown as shades of grey. Arcs show the combination of cytokine reactivities.

Deconvolution of cytokine combinations showed that on the day of superinfection challenge 6/6 completely protected animals had circulating SIV-specific quadruple cytokine expressing CD8^+^ cells at a frequency of >0.02% compared to only 1/6 partially protected macaques (ρ = 0.015; Fisher’s exact test). Differences in median frequencies of maximally polyfunctional CD8^+^ T cells between the groups did not reach statistical significance (ρ = 0.065; Mann-Whitney rank sum test) due to the outlier E71 (Fig 6). Similarly, IFN-γ + TNF-α dual positive CD8^+^ T cells were absent or below 0.02% in partially protected animals whereas in the completely protected group 5/6 macaques had frequencies markedly above 0.02% (ρ = 0.015; Fisher’s exact test) with a significantly elevated median frequency (ρ = 0.026; Mann-Whitney rank sum test). Significant differences were not seen for any cytokine combination 130 days after challenge (Fig 7). No significant differences were seen between partially and completely protected groups in the frequencies of circulating SIV-specific CD4^+^ T cells expressing individual cytokine combinations at either the day of superinfection challenge (S7 Fig) or 130 days after challenge (S8 Fig).

### Nef-specific polyfunctional CD8^+^ T cells were detected in mesenteric lymph nodes regardless of protection status

**Fig 8:**
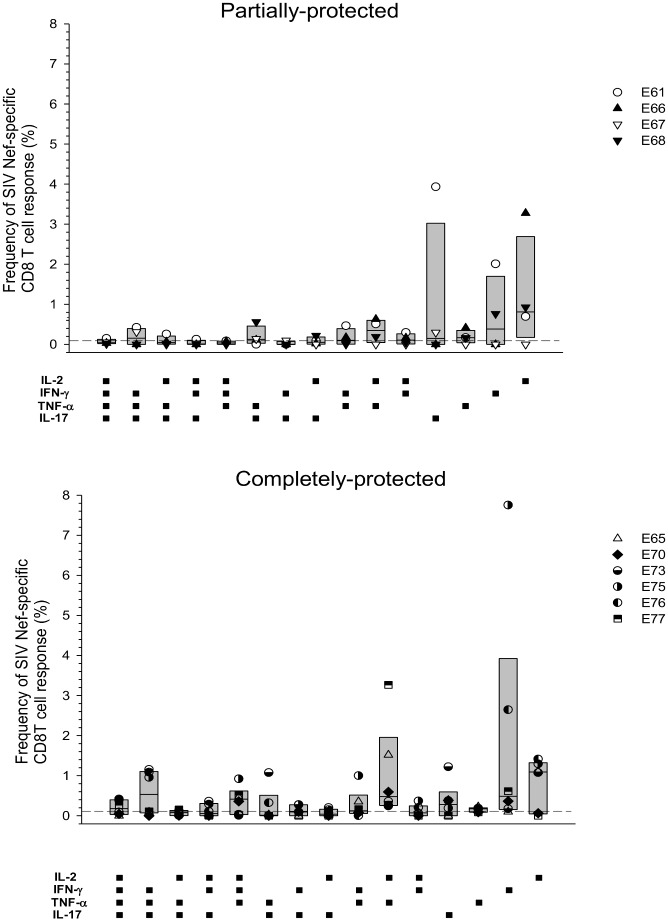
Comparison of the frequency of Nef-specific CD8^+^ T cells in mesenteric lymph nodes 130 days after superinfection challenge with respect to protection status. Cell populations were determined by multi-parametric flow cytometry following stimulation of MNC with SIV-Nef peptide pools. Background responses detected in medium alone control samples were subtracted for every combination of cytokines and a cut-off of >0.01% after background subtraction was used as the threshold for positive reactivity (dashed line). Box plots show the 25^th^ and 75^th^ percentiles and median (solid line) and mean (dotted line) for each cytokine combination.

Although it was not possible to discern protection status-specific differences in circulating CD8^+^ T cell responses 130 days after wt-challenge, responses in lymphoid tissue may be more informative. Mononuclear cells extracted from mesenteric lymph nodes at necropsy were stimulated *in vitro* with a pool of Nef unique region-specific peptides. SIVrtTA and SIVmac239Δ*nef* vaccine strains do not produce Nef protein, whereas the SIVmac239 challenge virus expresses full-length Nef. Surprisingly, poly and mono-functional CD8^+^ T cells were detected regardless of protection status (Fig 8). Although statistically different frequencies of functional cells were not detected between the groups, there was a trend towards higher reactivity in completely protected animals.

### Nef antigen was detected in spleen from all SIVrtTA vaccinates but not from macaques vaccinated with SIVmac239Δ*nef*

**Fig 9:**
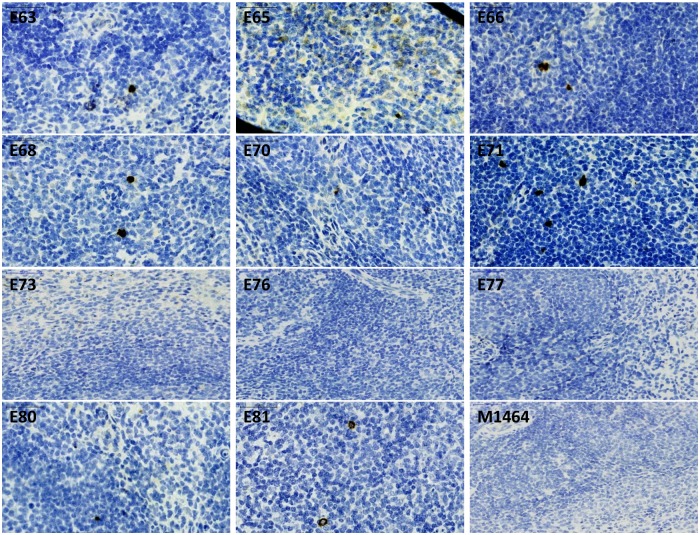
Evidence of wild-type infection the spleen of protected and partially protected macaques. Foci of SIV positive wild-type SIVmac239 infection in all SIVrtTA vaccinates compared with Group C macaques vaccinated with SIVmac239Δ*nef* (E76 and E77) stained with KK77 monoclonal antibody that detects wild-type Nef only. Distribution of wild-type SIV positive cells in Group D (E80, E81) and a naïve, uninfected macaque (M1464) are shown for comparison. Staining for protected macaques E65 and E70 is more diffuse and sporadic compared to naïve wild-type SIVmac239 challenge control staining. Magnification factor X40.

Sections of spleen from vaccinates, naïve challenge controls and unchallenged macaques were stained with KK77 monoclonal antibody specific for Nef (Fig 9). Positive cells were detected in partially-protected SIVrtTA vaccinates as well as fully-protected macaques E65 and E70. In contrast, macaques of Group C vaccinated with SIVmac239Δ*nef* were indistinguishable from negative controls. Although clearly detectable staining for Nef was present in E65, the staining pattern was more diffuse with occasionally identifiable foci of positive cells, as distinguished from productively infected macaques which were partitioned into the partially protected group.

## Discussion

The reported breadth and duration of protection conferred in macaques following vaccination with live attenuated SIV has many of the features required of an effective vaccine against HIV/AIDS. Understanding the mechanisms of protection may allow the informed design of intrinsically safe vaccines. Earlier attempts to improve the safety profile of live attenuated SIV by introducing multiple attenuating mutations revealed that the degree of protection was inversely proportional to the degree of attenuation [11]. Hence, it was perhaps not unexpected that SIV clones molecularly engineered to be limited to a single round of replication conferred only limited protection compared with more vigorously replicating attenuated vaccine strains [23, 33]. The development of SIVrtTA with potential to be temporally modulated for replication *in vivo* provides a novel tool to further dissect the processes of protection elicited by live attenuated SIV. Previously, we reported this novel virus to replicate *in vivo* and being fully infectious in rhesus macaques, with the ability to disseminate to lymphoid tissues and elicit a range of immunological responses including reversible changes in the frequency of memory T cell subsets dependent upon the withdrawal of dox [26].

Here, we report that vaccination with SIVrtTA confers protection against homologous wild-type challenge, in some cases similar to the ‘gold-standard’ SIVmac239Δ*nef* vaccine. However, levels of protection were variable. Full or complete protection (based on absence of a wild-type post-challenge viraemia) was associated with a prolonged shoulder of persisting SIVrtTA vRNA signal in plasma during the dox-on period rather than the later modulation of replication in lymphoid tissues (occult replication) mediated by the administration of dox. This aberrant viraemic profile may be dependent upon intrinsic host factors, for example the availability of alternative secondary receptors, or mutational events in the vaccine virus. Notably, mutations in rtTA which do not affect dox-dependence were detected only in the fully protected macaques and may have contributed to the fitness of SIVrtTA *in vivo*. Interestingly, a similar virological profile was seen also in one animal vaccinated with SIVmac239Δ*nef*. Despite the replicative fitness cost introduced by the dox-dependent regulatory elements, the remaining animals vaccinated with SIVrtTA demonstrated significant protection from wild-type SIVmac239 challenge, as breakthrough of challenge virus was at lower levels than naive challenge controls with reduced lymphoid virus sequestration. These results support the observation that in the SIV/macaque model, and in common with other live attenuated vaccines, a defining feature of efficacy is related to the ability of the vaccine virus to replicate in the early phases of vaccination and in addition, suggest that limited acute phase replication may be compensated by subsequent persistence.

SIVrtTA shows absolute dependency upon dox for its replication *in vitro* [24, 25] and as we have shown previously, dox status influences the T_EM_ circulating pool [26]. Nevertheless, we have not formally directly demonstrated that dox status completely controls replication *in vivo* in all anatomical compartments. Whilst we consider that loss of dox-dependency is unlikely, given the lack of mutations in the known critical sites, future experiments could include challenge of naïve macaques in the absence of administration of doxycycline.

Application of discriminatory PCR assays able to unravel the relative contributions of each virus to detectable PCR signals was a critical component of this study. These assays unequivocally established that plasma RNA following challenge of Group C animals, and of fully protected SIVrtTA vaccinates E70 and E65 was vaccine-virus specific. Moreover, this was corroborated by analysis of CA-RNA in lymphoid tissues at the termination of the study. A hallmark of complete vaccine protection appeared to be the persistent replication of vaccine virus in lymphoid tissue. Surprisingly however, only a very low level of vaccine CA-RNA was detected in a single tissue of macaque E75 suggesting there may have been persistence elsewhere such as the gut and/or vaccine generated immunity had cleared infection to limits below detection at least in the tissues examined. The mechanism for the persistent low-level replication of SIVrtTA in the absence of dox in macaque E65 is unknown. As we have reported previously, low levels of vRNA have been detected by *in situ* hybridisation in small intestine from SIVrtTA-infected rhesus macaques following dox withdrawal [26]. Therefore, we are unable to formally exclude the possibility that dox-dependency *in vivo* is conditional.

It was notable that where breakthrough virus was detected in lymphoid tissues of Group B animals, maintained on dox throughout the experiment, there was no evidence of residual vaccine virus. We reported previously that proviral DNA was detected pre-challenge in the spleen, PLN and MLN of animals maintained on dox although concentrations were lower than in macaques vaccinated with SIVmac239Δ*nef* [26]. Presumably, given the fitness disadvantage, any extant replicating SIVrtTA was displaced by the challenge wild-type virus.

Although in this study we were unable to definitively address whether persisting vaccine virus replication in lymphoid tissue is an absolute requirement for complete protection because of the reduced replication of SIVrtTA, the opportunity was available nonetheless to compare T memory cell frequencies and cellular immune responses in partially and completely protected groups. The T memory cell results showed a strong association with protection status, which in most analyses reached statistical significance. The complete loss of these associations when analysis was done 130 days after challenge is striking, particularly in (1) the polarisation of CD4^+^ memory T cells toward the T_CM_ phenotype regardless of protection status and (2) the changes in proportions of CD8^+^ memory T cells in completely protected animals. Although not reaching statistical significance due to outliers there was a clear trend for reduction in the number of CD8^+^ T_EM_ with a concomitant increase in T_CM_. This latter effect probably reflects a reduction in on-going antigen re-stimulation *in vivo* at this time and/or a redistribution of T_EM_ to tissue compartments. We did attempt analysis in gut tissues taken *post mortem*; however, cell recovery was poor making interpretation of flow cytometric data unreliable.

Despite the reported lack of association between responses detected in the blood and subsequent protection [10, 12, 6], we identified a statistically significant association between high frequencies of global T_EM_ in peripheral blood at the time of challenge and outcome. Moreover, total frequencies of SIV-specific polyfunctional CD8^+^ T cells were significantly higher in macaques exhibiting complete protection, compared with partially protected macaques, on the day of challenge. Interestingly however, macaque E65, which demonstrated continuous very low-level replication of SIVrtTA in the absence of dox, failed to show this association, perhaps suggesting that other factors may be associated with complete protection in this animal. Further analysis of cytokine combinations revealed that CD8^+^ memory T cells with quadruple cytokine staining and cells staining for IFN-γ and TNF-α were present at higher frequency in complete protection compared with the frequencies in partially protected animals. Interestingly, the one macaque that did not have detectable dual-stained CD8^+^ T cells, E70, had an exceptionally high frequency of quadruple staining cells. Clearly, this analysis represents only a fraction of the total picture, since proteome-wide expansion of T cells was not performed and only 4 cytokines were analysed.

ICS staining for IL-17 was included in the present study since perturbations in CD4^+^ and CD8^+^ IL-17-staining cells in both the periphery and mucosal compartments reportedly reflect SIV-induced changes in disease status [34–36] and therefore could be a useful marker particularly in animals that may become dually-infected after challenge with virulent virus (*i*.*e*. may indicate sparing from disease progression). Several animals displayed unexpectedly high IL-17 positivity either before or following superinfection challenge. The reasons for this are not known; however, it is worth pointing out that these results were obtained in the context of infection with a novel SIV construct and it is possible that in certain genetic backgrounds this virus stimulates a strongly regulatory T cell phenotype.

Analysis of SIV-specific CD8^+^ T cell frequencies in mesenteric lymph nodes did not reveal a difference between completely and partially protected animals; however, it did reveal evidence of a challenge virus footprint. The Nef-specific T cell responses seen could only be stimulated by wild-type virus challenge. As Nef is not a structural component of the virus, this would require *de novo* synthesis of Nef in infected cells. The absence of Nef-staining in the spleen of SIVmac239Δ*nef* vaccinated animals is consistent with the notion that the mechanism of complete protection from wild-type virus challenge operates through early clearance of challenge virus; whereas in partially-protected animals T cells may suppress wild-type virus replication rates relative to those in vaccine naïve animals. It was however surprising that a low level of Nef staining was detected in the apparently completely-protected SIVrtTA vaccinated animals. Thus, although by the criteria of RNA detection and Gag-specific antibody responses post-challenge these animals appeared to be completely protected, they should perhaps be considered falling into an intermediate category between completely and partially protected. Clearly, however, these macaques were protected from overt, productive superinfection.

In this regard the timing between exposure to wild-type virus and recovery of tissues at autopsy for analysis may be critical. In this study a relatively long period (20 weeks) was allowed to elapse from time of wild-type challenge to autopsy, which is likely to have allowed sufficient time for a response to wild-type virus to be generated but where the virus was no longer detected at termination. In such a scenario, the challenge virus is likely to have been present at some level but which had been subsequently cleared by host T cell responses to wild-type virus infection reflecting previous reports in the literature where much earlier sampling for virus post-challenge (*eg* 14 days after wt challenge) resulted in detection of virus in tissues at necropsy but the overall virological phenotype was that of protection [37]. The likely role of T cells in this protection has been further demonstrated by CD8 T cell depletion experiments where control of the replication of both the challenge and vaccine viruses have been linked to a CD8 T cell response [38, 39].

Recently reported detailed analysis of immune responses and deep sequence characterisation of SIVmac239Δ*nef* post-vaccination indicated that there is a shift following early, rapid virus escape due to immune pressure to variable regions targeted during the acute phase to a re-focussed immunological response to more conserved epitopes [40]. However, the level of sub-clinical antigenic drive required to deliver such an anentropic state requires clarification, perhaps also in the face of host responses to the vaccine virus, since it was also noted that macaques with undetectable plasma viraemia experienced ongoing sequence evolution of the vaccine virus. It is perhaps noteworthy that in our study we observed distinct sequence changes in the rtTA gene rescued from viral RNA in plasma, taken as a measure of recently replicating virus, in the two SIVrtTA protected macaques (E65, E70) during the early, post-acute phase of virus replication which further marked these macaques out as being virologically distinct from the other SIVrtTA vaccinates. Hence, viral evolution as a driver for improved virological fitness *in vivo* during the post-acute phase appears to have had a marked effect in terms of the overall protection status conferred on these two macaques. SIVrtTA replication in macaques will also probably induce immune responses not only against viral proteins but also against rtTA itself [41]. Therefore, it is plausible that the observed amino acid changes mediate a mechanism of immune-escape of the rtTA protein, which would likely improve persistent virus replication, but this was not formally investigated.

Hence, SIVrtTA vaccination of Indian rhesus macaques appears on the cusp of delivering potent vaccine protection. If SIVmac239Δ*nef*-induced protection correlates with an expanded T cell anentropy to highly conserved epitopes with an associated increased depth of response generated over time, this likely explains the relatively poor ability of a ‘one-hit’ vaccine response, such as single cycle SIV to ensure long-lived vaccine protection. Compensations in vaccine replication appear important in conferring protection mediated by SIVrtTA, although whether these are sufficient to explain features of early protection from heterologous challenge, for example, remains unclear. Highly attenuated viral vaccines such as SIVmacΔ4 [11] which have a reduced replication potential *in vivo*, but which fail to persist, exhibit an intermediate protection profile. Hence the ability of SIVrtTA to exhibit low, continuous replication provides a clear advantage compared to these approaches. Lack of an increased magnitude of SIV-specific CD8 T cell responses in lymph nodes correlating with proposed mechanisms of protection for cellular responses at key sites of virus replication in the body [40], suggest that the role of CD8 T cells in this mode of vaccine protection is far from resolved, whereby a higher viral replication in turn leads to higher CD8 T cells responses in lymphatic tissue [10].

On the face of it our data appears to strongly support the view that CD8^+^ polyfunctional T_EM_ are critical in protective immunity induced by live attenuated SIV as suggested by Fukazawa *et al* [10] for lymph node responses. However, technical limitations precluded the ability to assign ICS responsiveness specifically to memory phenotype in our study, and as in other studies, our observations remain correlative. Indeed, antibody responses to Gag p27 before and after vaccine challenge are also predictive of outcome but are unlikely of mechanistic significance. If the current paradigm of live attenuated vaccine protection is correct, it must also explain why superior responses in the host that prevent viral infection are established in the same host where host control of the vaccine is poorest. This counterintuitive observation requires a cogent answer irrespective of localisation of the vaccine virus *e*.*g*. in T-follicular helper cells which may be subject to immune privilege, or magnitude and breadth of measurable immune responses such as CD8 T cell responses which are potentially capable of targeting and controlling both vaccine and challenge viruses, yet the vaccine virus is able to persist at these key sites.

Taken together, our data provide further insight into the highly dynamic process of live attenuated SIV vaccine outcomes where the replicative properties and persisting nature of the vaccine virus appear crucial to vaccine efficacy. SIVrtTA provides a novel tool in our armoury to understand more fully processes of occult and patent virus replication at niche anatomical sites where issues of viral latency and persistence are crucial in understanding retrovirus and immune interactions.

## Materials and Methods

### Ethics statement

Non-human primates were used in strict accordance with UK Home Office guidelines, under a licence granted by the Secretary of State for the Home Office which approved the work described. Animal work at NIBSC is governed by the Animals (Scientific Procedures) Act 1986 that complies with the EC Directive 86/609 and performed under licence (PPL 80/1952) granted only after review of all procedures in the licence by the NIBSC local Animal Welfare and Ethical Review Body. All study macaques were purpose bred and group-housed for the entire duration of the study, with daily feeding and access to water *ad libitum*. Given the limited availability of suitable macaques, age, sex and weight matching was not possible, nor central to the study outcome. Regular modifications to the housing area were made by husbandry staff including introduction of novel structures (*eg* swings and perching stations) and foodstuffs in novel manners to encourage foraging for food, to further enrich the study environment. The environmental temperature (15–24°C), was appropriate for macaques and rooms were subject to a 12 hour day/night cycle of lighting. Animals were acclimatised to their environment and deemed to be healthy by the named veterinary surgeon prior to inclusion on the study.

All animals were sedated with ketamine prior to bleeding or virus inoculation by venepuncture. Frequent checks were made by staff and any unexpected change in behaviour by individuals on study followed up, including seeking of veterinary advice where necessary. Regular blood evidence of incipient disease and veterinary advice were sought when persisting abnormalities detected. The study was terminated and all animals killed humanely by administering an overdose of ketamine anaesthetic prior to development of overt symptomatic disease. All efforts were made to minimise animal suffering, including provision of a high standard of housing quarters and monitoring of animal health and well-being and the absence of procedures not essential to the study.

### Study outline and viruses

16 UK purpose-bred Indian rhesus macaques (*Macaca mulatta*) were used in the study, in accordance with UK Home Office guidelines (Code of Practice 1988) and local ethical approval. The basic construction and mode of action of the SIV-rtTAΔ*nef* (SIVrtTA) vaccine, based on a SIVmac239 genetic backbone, is depicted in Fig 1A. In a challenge study experiment, eight macaques were inoculated intravenously with 5 x 10^3^ TCID_50_ SIVrtTA vaccine receiving 100mg daily oral dosing with dox. In four macaques (Group A), dox was removed eight weeks prior to SIVmac239 wild-type challenge. In the remaining four SIVrtTA vaccinates (Group B) dox dosing was maintained at 100 mg daily oral dosing. Group C comprised four macaques inoculated with 10^4^ TCID_50_ SIVmac239Δ*nef*. All vaccinates were challenged with wild-type SIVmac239 in addition to four additional macaques which served as naïve challenge controls (Group D). The study outline is summarised in Fig 1B. Veterinary procedures deployed the use of ketamine hydrochloride prior to sedate macaques. Plasma concentrations of dox were monitored *ex vivo* using a previously described assay [41].

### Host genetics

Macaques were genetically characterised for host MHC profiles, by Dr David Watkins (Univ. Wisconsin, S2 Table). Distribution of TRIM5α and TRIMcyp alleles was determined as previously described. *Mamu*7 represents macaques harbouring the TRIMcyp allele [42].

### Quantitative SIV RNA and DNA levels and differential PCR

Initial quantitative measures were made in peripheral blood using quantitative *gag*-based real-time PCR assays as previously described [6]. Plasma vRNA levels were determined for EDTA-treated plasma samples with a limit of detection of 50 SIV RNA copies/ml and SIV DNA levels on PBMCs with limit of detection one SIV DNA copy/100,000 cell equivalents. SIVrtTA-specific levels were determined using primers designed to amplify a region of the rtTA gene using PCR conditions comparable to those described for the total *gag* estimations against an rtTA plasmid containing unique sequences to the rtTA gene. SIVrtTA-specific amplification sequences were CGCCGTGGGCCACTT (forward), and CTTTCCTCTTTTGCTACTTGATGCT (reverse); internal rtTA probe sequence was FAM-CACTGGGCTGCGTATTGGAGGAACAG-BHQ1; primers and probes were used at 100nM concentrations. Wild-type SIVmac239-specific amplifications were made with CTCAGGACCAGGAATTAGATACC (forward), AAGGGTCATCCCACTGGGAAGT (reverse) and internal probe sequence FAM-TCCCTGTAAATGTATCAGATGAGGCACAGGAGG-BHQ1 targeting the *nef* gene. Primers were used at 100nM and probe at 120nM concentrations. Detection limits of virus-specific amplification in plasma were determined to be 100 RNA copies/ml with an amplification efficiency of >98%.

Cell-associated RNA determinations were made for SIVrtTA, SIVmac239Δ*nef* and wild-type SIVmac239 respectively by adapting a previously reported method [7]. Total RNA was isolated from spleen, mesenteric and peripheral lymph nodes using an RNeasy kit (Qiagen), subjected to on-column DNAase treatment in accordance with the manufacturers’ protocol. Virus-specific targets were amplified by one-step RT-PCR using 50ng total RNA input, adapting the SIVrtTA and SIVmac239 wild-type specific primers described above and employing those described previously in [43] for SIVmac239Δ*nef-*specific amplification as follows: cttaggagaggtggaagatggatactc (forward), CTTTTCTTTTATAAAGTGAGACCTGTTCC (reverse) and internal probe sequence FAM- CAATCCCCAGGAGGATTAGACAAGGGCTTG -BHQ1. Primers were used at 300nM and probe at 75nM. All CA-RNA determinations were made using normalised values of GAPDH, in co-amplification reactions as described in [7]. All amplifications were performed with Invitrogen Ultrasense kits with a thermoprofile of RT step 52°C for 30 mins; 10 mins at 95°C then 40 cycles of 95°C for 30 seconds and 60°C for 60 seconds. Limits of detection for SIVrtTA, SIVmac239Δ*nef*, wild-type SIVmac239 CA-RNA assays were determined as 50, 34 and 80 SIV RNA copies per 50ng total RNA. All CA-RNA quantitative PCR assays had an efficiency of >95%, typically 98–99% efficiency of amplification. The SIVrtTA assays were validated using a plasmid construct denoted rtTAV16 diluted to an extinction end-point in quantitative assays.

### Serology

Plasma anti-SIV p27 IgG responses were quantified by ELISA. Briefly, medium binding 96-well plates (Greiner, UK) were coated with 1μg/ml recombinant SIV p27 (CFAR, UK, Cat no: EVA664). Test plasma and standard positive and negative control samples were added to washed plates and bound IgG detected with goat anti monkey IgG-HRP (Serotec) followed by addition of substrate to induce a colour reaction in reactive samples.

### Immunophenotyping and intracellular cytokine staining

Memory phenotype and intracellular cytokine staining were performed separately in each sample per animal due to limitations of the flow cytometry capability available. Peripheral blood lymphocytes (PBL) were isolated using Percoll gradient centrifugation and mesenteric lymph node mononuclear cells (MNC) were isolated by mechanical disaggregation of tissue. To delineate memory T cell subsets, PBL were simultaneously surfaced stained with anti-CD3-V500 (clone SP32, BD Horizon), anti-CD4-V450 (clone L200, BD Horizon), anti-CD8-APCCy7 (clone SK1, BD Biosciences), anti-CD95-PECy7 (DX2, BioLegend), anti-CD28-PerCP-Cy5.5 (eBiosciences), and anti-CCR7-FITC (R&D systems). Gates on lymphocyte subpopulation were defined as central memory CD8^+^C95^+^CD28^+^CCR7^-^ and CD8^+^CD95^+^CD28^-^ CCR7^-^ as effector memory.

SIV-specific T cell responses were determined by cytokine production after incubation with 5 μg/ml of either SIV Gag, Tat, Rev or (for MLN MNC additionally Nef peptides from the *nef* -unique coding region) (15mers overlapping 11 residues, CFAR/NIBSC, Potters Bar, UK) plus 10 μg/ml CD49d, 50μg/ml anti-CD28, Golgi Stop (10ng/ml, BD), and incubated at 37°C in a 5% CO_2_ environment with RPMI 1640/10% FCS for 14h. Stimulated cells were surfaced stained for CD3, CD4 and CD8, permeabilised (Fix and Perm kit, Caltag), and then stained for intracellular cytokine detection with anti-IFNγ-PErCPCy5.5 (clone B27), anti-IL-2-PE (MQ1-17H21, eBiosciences), anti-TNF-α-APC (MAB11, eBiosciences) and anti-IL-17-Pacific Blue (BioLegend). Polyfunctional T cells were determined by a gating strategy as shown in the representative plots (S9 Fig). In detail, within CD4 and CD8 subsets, distribution of TNF-α and/or IL-2 producing cells were specified using contour FACs profile quadrants. Each quadrant within these cell populations were sequentially analysed for IFN-γ and/or IL-17 production in combinatory plots. For group comparisons (partial versus complete), total frequencies of ICS-stained cells were derived by adding mono, bi, tri and quadruple functional frequencies for each animal. The relative distribution of the cytokine producing cells in each animal was summarised in pie charts using SPICE software.

All peripheral and tissue derived mononuclear cells were acquired and analysed using a BD Canto II flow cytometer (BD Immunocytometry) with FACS DIVA software as described previously [26].

### Graphing and statistical analysis

Graphing and associated statistical analyses, as specified, were performed using Sigma Plot 11 (Systat Software, Inc.). Kruskal-Wallis analyses of variance with Dunn’s post-hoc test were determined using the Minitab version 17 software. In addition, analysis and graphical representation of cytokine production were conducted using the data analysis programme Simplified Presentation of Incredibly Complex Evaluations (SPICE, version 5.3) provided by M. Roederer, National Institutes of Health, Bethesda, MD.

### Immunohistochemistry

Immunochemical staining for Nef was performed with the KK77 antibody (CFAR; ARP3093) which is an IgG2a isotype raised to recombinant SIVmac251 Nef and which detects wild-type Nef only, using protocols as previously described [7].

## Supporting Information

S1 Fig Comparison of vaccine-only vRNA loads between *mamu*A-01 positive and negative vaccinates. Dynamics of SIV RNA levels (expressed as SIV RNA copies/ml plasma) of SIVrtTA or SIVmac239Δ*nef* vaccinates stratified according to *mamu* A-01 status in macaques followed out to immediately prior to wild-type SIVmac239 challenge. 1/4 *mamu* A-01 positive macaques were completely protected compared with 5/8 *mamu* A-01 negative macaques.
(TIF)

S2 Fig Quantitative vDNA measures. Wild-type SIVmac239-specific DNA signals detected in a wide range of tissues compared for SIVrtTA vaccinates (Goups A and B), SIVmac239Δ*nef* vaccinates (Group C) and challenge controls (Group D) 20 weeks after administration of wild-type SIVmac239 challenge. Nef-specific signals were expressed as SIV DNA copies per 100,000 mononuclear cells (MNCs). Tissues sampled were spleen, mesenteric lymph nodes (MLN), peripheral lymph nodes (PLN), thymus, small and large intestines and brain as indicated.
(TIF)

S3 Fig Comparison of longitudinal anti-Gag p27 IgG responses in fully and partially protected vaccinates. Anti-SIV p27 responses are shown prior to and post wild-type SIVmac239 challenge. None of the protected macaques (E65, E70, E73, E75, E76, E77) displayed a boosted antibody response to SIV Gag p27 antigen. All partially protected macaques (E61, E63, E66, E67, E68, E71) exhibited a boosted anti-SIV p27 response.
(TIF)

S4 Fig Mutations observed in the U3 LTR promoter region upon *in vivo* replication of SIVrtTA. In SIVrtTA, two tetO sequences had been inserted between the NFκB and Sp1 binding sites in the U3 domain of the LTR promoter. In previous *in vitro* culture experiments, continuous serial passaging of SIVrtTA in CEMx174 cells had resulted in triplication of a short region including the NFκB binding site and one tetO element, which was followed by deletion of upstream U3 sequences. This optimized SIVrtTAopt configuration was present in the SIVrtTA variant used in the current vaccination study. Sequencing of SIVrtTA RNA recovered from plasma of macaques at several times after vaccination revealed the frequent deletion of one of the NFκB-tetO repeats.
(TIF)

S5 Fig Mutations observed in rtTA do not affect transcriptional activity. 293T cells were transfected with a plasmid expressing wild-type (V16) or mutant rtTA and a promoter-reporter plasmid in which expression of firefly luciferase is controlled by the SIVrtTA LTR promoter [44]. After culturing the transfected cells with 0 to 100 ng dox ml^-1^ for 48 h, the intracellular luciferase level (RLU) was measured as previously described [25].
(TIF)

S6 Fig Analysis of total frequencies of SIV-specific mono and polyfunctional circulating T cells 130 days after superinfection challenge. Results for CD4^+^ cells (a) and for CD8^+^ cells (b) are shown for individual macaques and as groups based on superinfection protection status. Total frequencies were derived by addition of mono, bi, tri and quadruple functional cells for each peptide pool tested. Box plots show median and mean values with 25^th^ and 75^th^ percentiles. Statistically significant differences for groups are shown as ρ values determined by Mann-Whitney rank sum test.
(TIF)

S7 Fig Frequency and distribution of SIV-specific CD4^+^ peripheral blood T cells on the day of superinfection challenge with respect to protection status. Cell populations were determined by multi-parametric flow cytometry following stimulation of PBMC separately with SIV-Gag, Rev and Tat peptide pools. Background responses detected in medium alone control samples were subtracted for every combination of cytokines and a cut-off of >0.01% after background subtraction was used as the threshold for positive reactivity (dashed line). Frequencies were derived by addition of results for Gag, Rev and Tat. Box plots show the 25^th^ and 75^th^ percentiles and median (solid line) and mean (dotted line) for each cytokine combination (a & c). Proportionate functionality for each macaque (b & d) is shown as a pie chart, with quadruple positivity shown in black and triple to mono positivity shown as shades of grey. Arcs show the combination of cytokine reactivities.
(TIF)

S8 Fig Frequency and distribution of SIV-specific CD4^+^ peripheral blood T cells 130 days after superinfection challenge with respect to protection status. Cell populations were determined by multi-parametric flow cytometry following stimulation of PBMC separately with SIV-Gag, Rev and Tat peptide pools. Background responses detected in medium alone control samples were subtracted for every combination of cytokines and a cut-off of >0.01% after background subtraction was used as the threshold for positive reactivity (dashed line). Frequencies were derived by addition of results for Gag, Rev and Tat. Box plots show the 25^th^ and 75^th^ percentiles and median (solid line) and mean (dotted line) for each cytokine combination (a & c). Proportionate functionality for each macaque (b & d) is shown as a pie chart, with quadruple positivity shown in black and triple to mono positivity shown as shades of grey. Arcs show the combination of cytokine reactivities.
(TIF)

S9 Fig Representative plots showing the gating strategy used to derive frequencies of intracellular cytokine stained CD4^+^ and CD8^+^ PBMC. CD4 and CD8 subsets were defined via CD3. Within these subsets the distribution of TNF-α and/or IL-2 producing cells were specified using contour FACs profile quadrants. Each quadrant within these cell populations were sequentially analysed for IFN-γ and/or IL-17 production in combinatory plots.
(TIF)

S1 Table Mutations in SIVrtTA observed upon *in vivo* replication. SIVrtTA RNA was recovered from plasma at several times after vaccination. The rtTA region was analyzed by RT-PCR and direct sequencing of the PCR product (population sequence). The LTR region was analyzed by RT-PCR, TA-cloning of the PCR product and sequencing of 9 to 12 TA clones. Silent and non-silent codon changes observed in the rtTA coding region are shown. The frequency at which mutations are observed in the LTR region is indicated between brackets (n.a., not analyzed).
(DOCX)

S2 Table Mamu MHC profiles for 4 class A and 5 class B MHC alleles for all study vaccinates and challenge controls. TRIM5/cyp status for each macaque was determined as indicated. X indicates inability to type this macaque.
(DOCX)
