# Supplementary figures and images for: Role of Occult and Post-acute Phase Replication in Protective Immunity Induced with a Novel Live Attenuated SIV Vaccine
Source: PLoS Pathog. 2016 Dec 21;12(12):e1006083. doi: 10.1371/journal.ppat.1006083 (PMC5176322; doi:10.1371/journal.ppat.1006083)

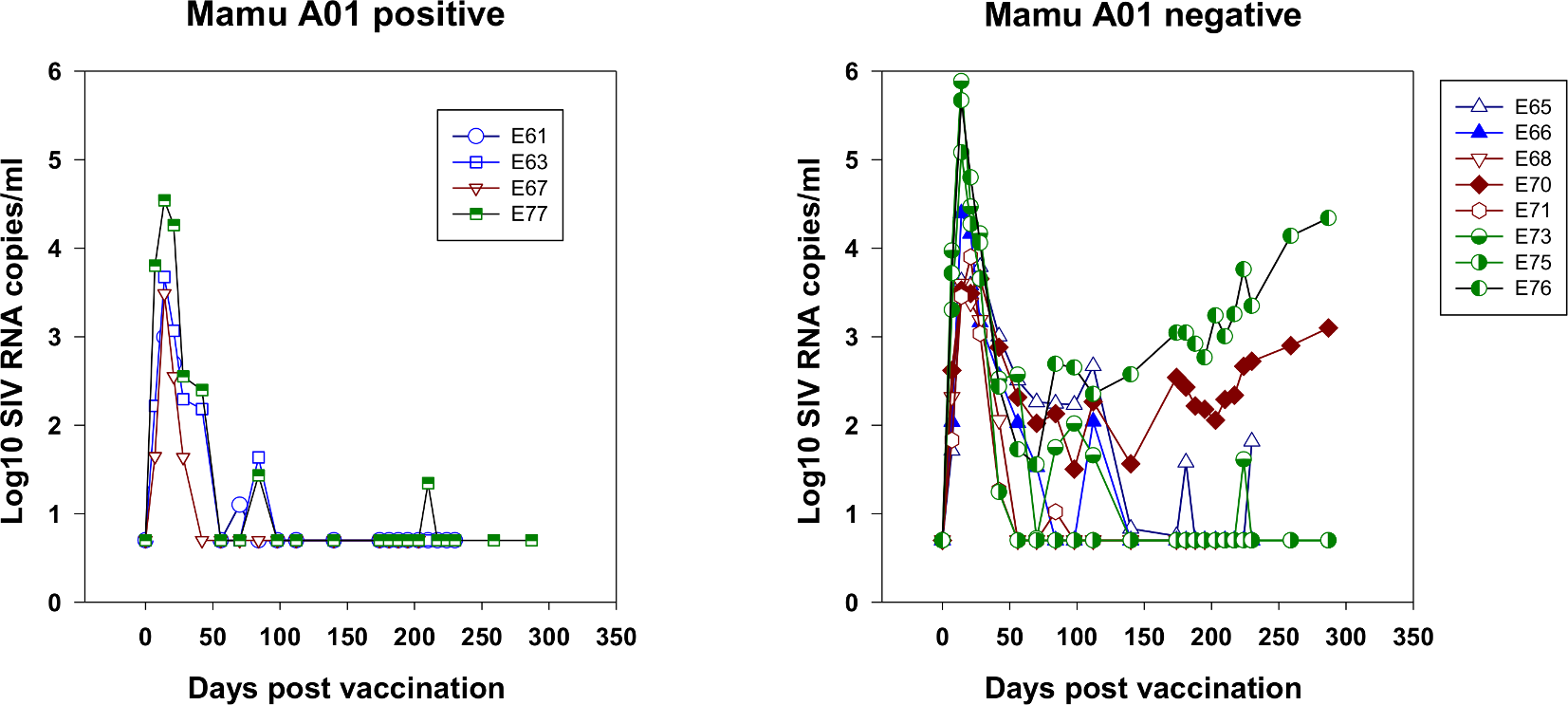

Supplement: S1 Fig — Dynamics of SIV RNA levels (expressed as SIV RNA copies/ml plasma) of SIVrtTA or SIVmac239Δnef vaccinates stratified according to mamu A-01 status in macaques followed out to immediately prior to wild-type SIVmac239 challenge. 1/4 mamu A-01 positive macaques were completely protected compared with 5/8 mamu A-01 negative macaques. (TIF) [file ppat.1006083.s001.tif]

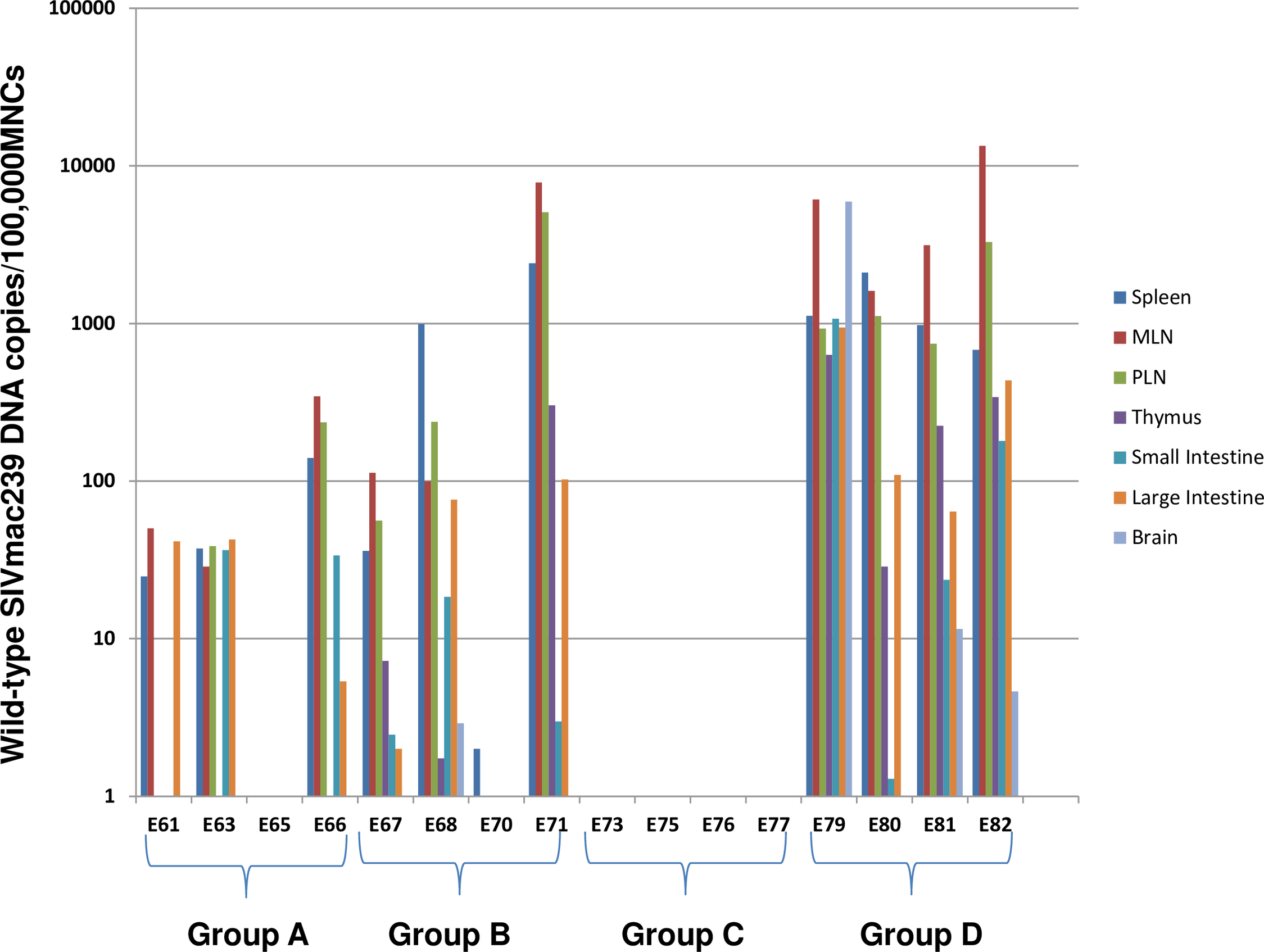

Supplement: S2 Fig — Wild-type SIVmac239-specific DNA signals detected in a wide range of tissues compared for SIVrtTA vaccinates (Goups A and B), SIVmac239Δnef vaccinates (Group C) and challenge controls (Group D) 20 weeks after administration of wild-type SIVmac239 challenge. Nef-specific signals were expressed as SIV DNA copies per 100,000 mononuclear cells (MNCs). Tissues sampled were spleen, mesenteric lymph nodes (MLN), peripheral lymph nodes (PLN), thymus, small and large intestines and brain as indicated. (TIF) [file ppat.1006083.s002.tif]

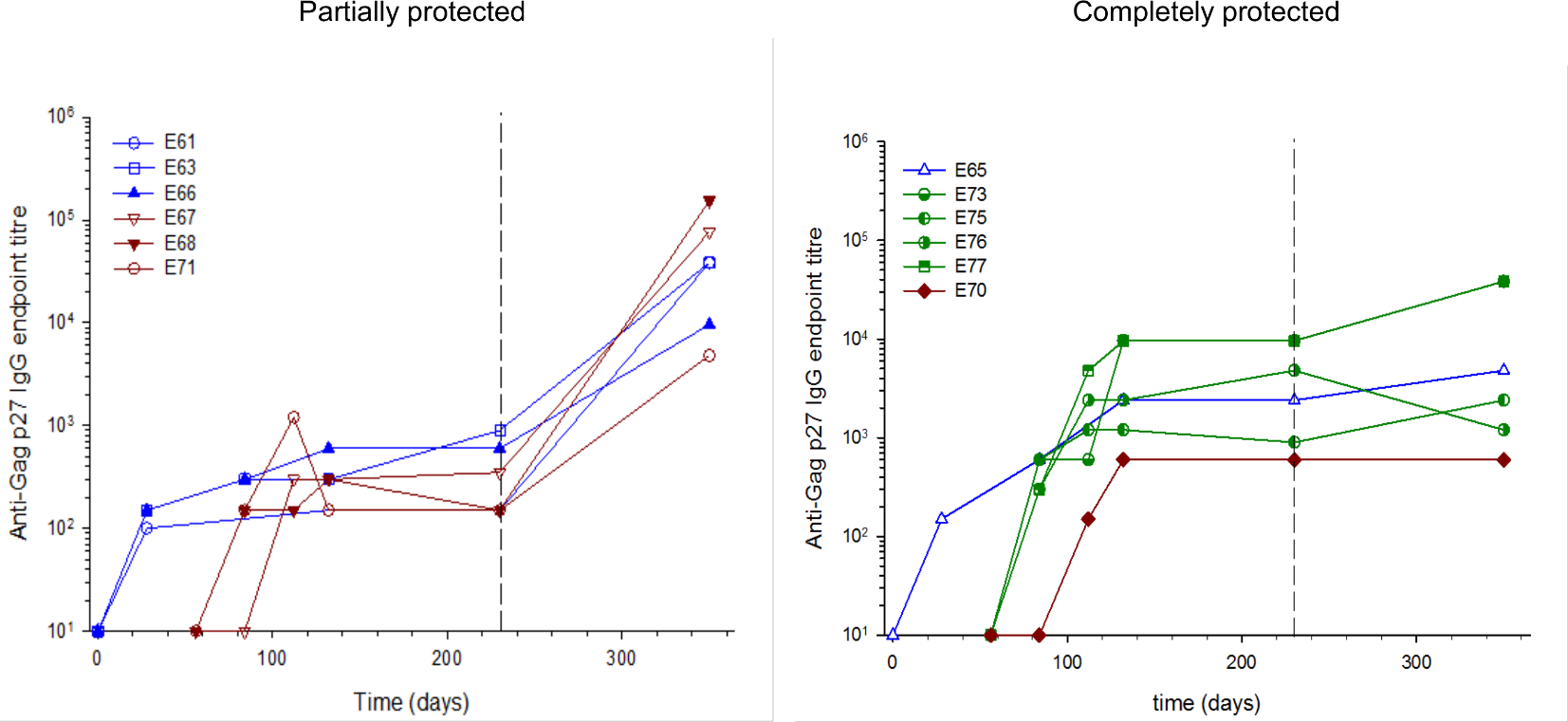

Supplement: S3 Fig — Anti-SIV p27 responses are shown prior to and post wild-type SIVmac239 challenge. None of the protected macaques (E65, E70, E73, E75, E76, E77) displayed a boosted antibody response to SIV Gag p27 antigen. All partially protected macaques (E61, E63, E66, E67, E68, E71) exhibited a boosted anti-SIV p27 response. (TIF) [file ppat.1006083.s003.tif]

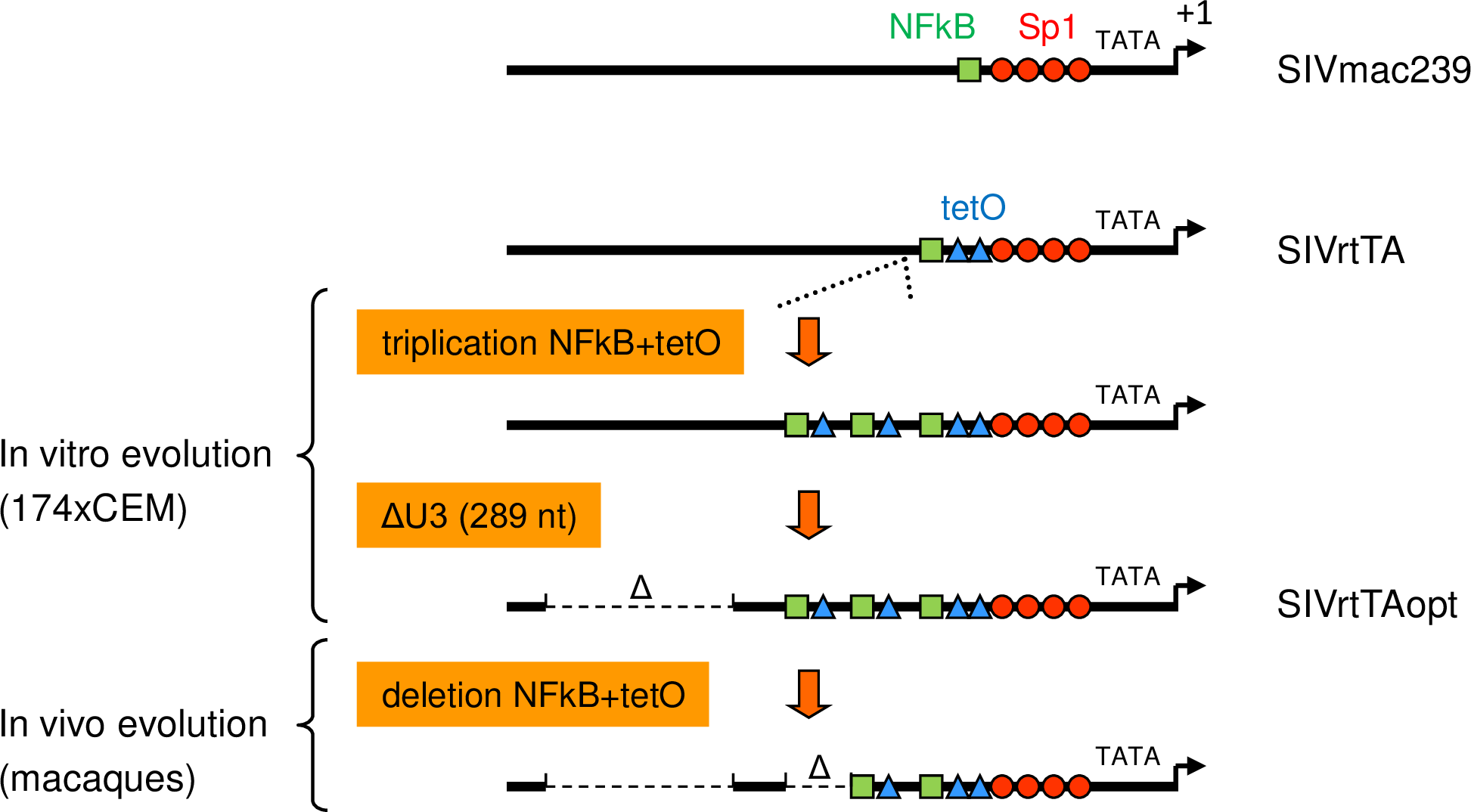

Supplement: S4 Fig — In SIVrtTA, two tetO sequences had been inserted between the NFκB and Sp1 binding sites in the U3 domain of the LTR promoter. In previous in vitro culture experiments, continuous serial passaging of SIVrtTA in CEMx174 cells had resulted in triplication of a short region including the NFκB binding site and one tetO element, which was followed by deletion of upstream U3 sequences. This optimized SIVrtTAopt configuration was present in the SIVrtTA variant used in the current vaccination study. Sequencing of SIVrtTA RNA recovered from plasma of macaques at several times after vaccination revealed the frequent deletion of one of the NFκB-tetO repeats. (TIF) [file ppat.1006083.s004.tif]

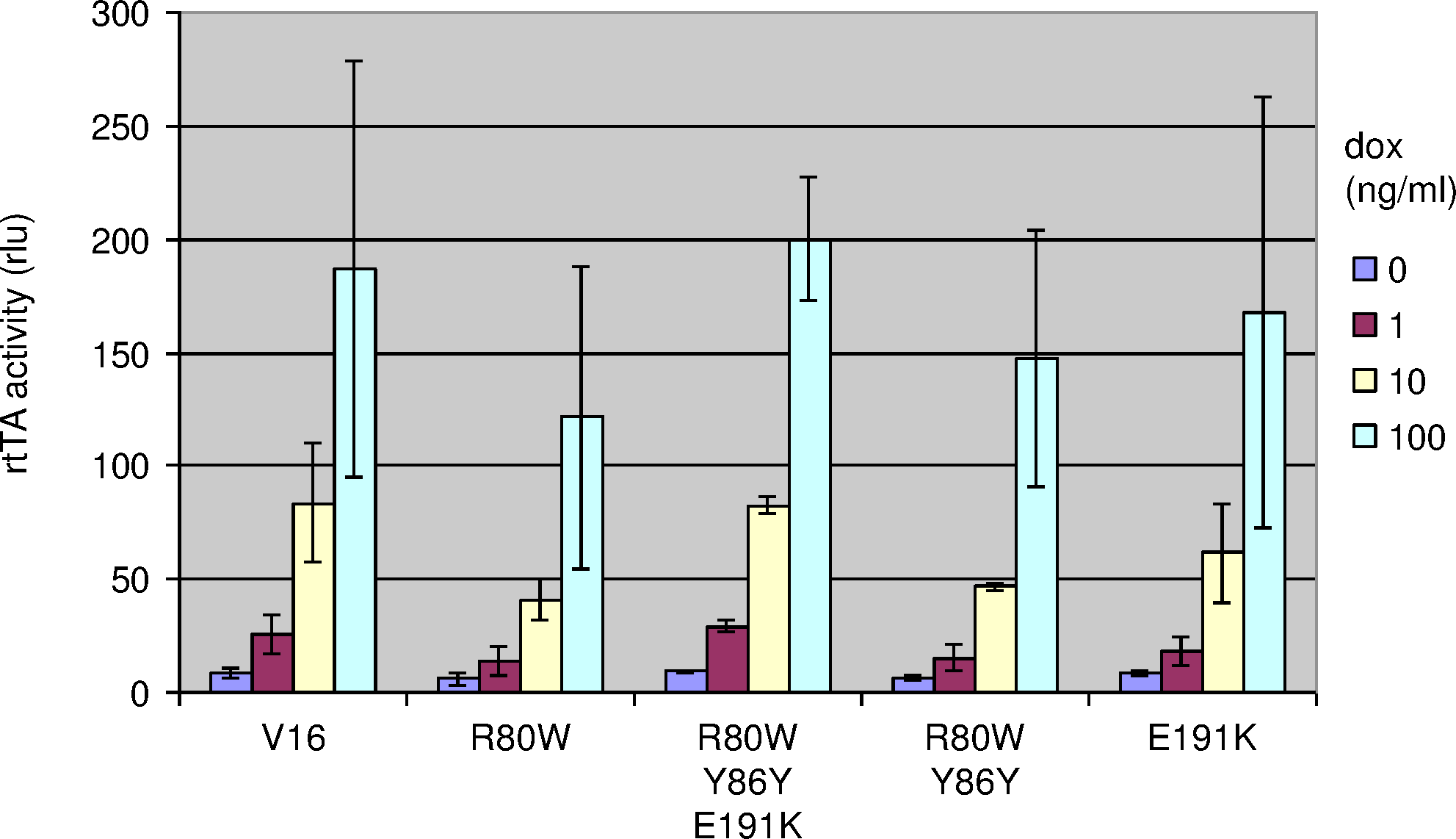

Supplement: S5 Fig — 293T cells were transfected with a plasmid expressing wild-type (V16) or mutant rtTA and a promoter-reporter plasmid in which expression of firefly luciferase is controlled by the SIVrtTA LTR promoter [44]. After culturing the transfected cells with 0 to 100 ng dox ml-1 for 48 h, the intracellular luciferase level (RLU) was measured as previously described [25]. (TIF) [file ppat.1006083.s005.tif]

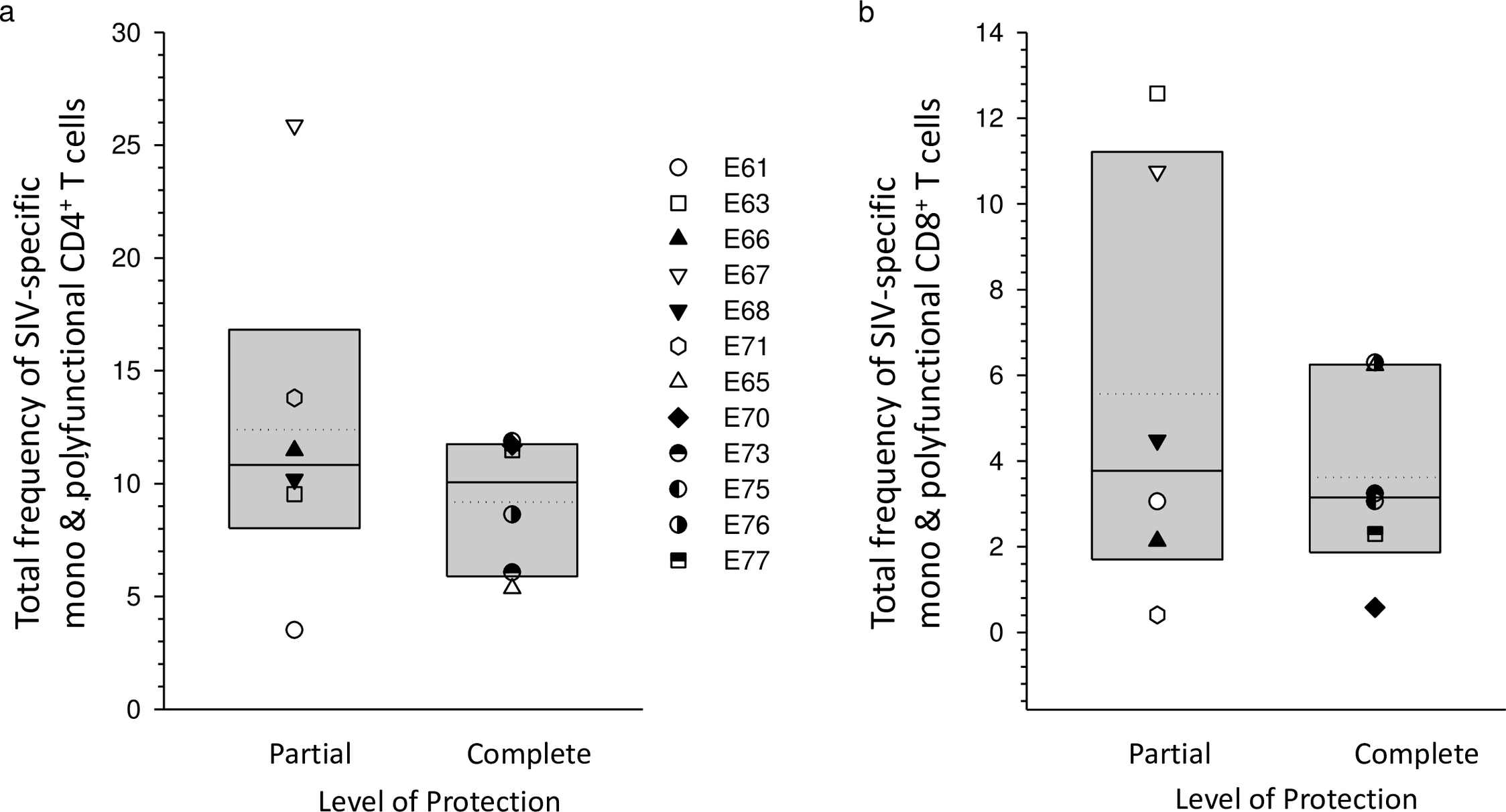

Supplement: S6 Fig — Results for CD4+ cells (a) and for CD8+ cells (b) are shown for individual macaques and as groups based on superinfection protection status. Total frequencies were derived by addition of mono, bi, tri and quadruple functional cells for each peptide pool tested. Box plots show median and mean values with 25th and 75th percentiles. Statistically significant differences for groups are shown as ρ values determined by Mann-Whitney rank sum test. (TIF) [file ppat.1006083.s006.tif]

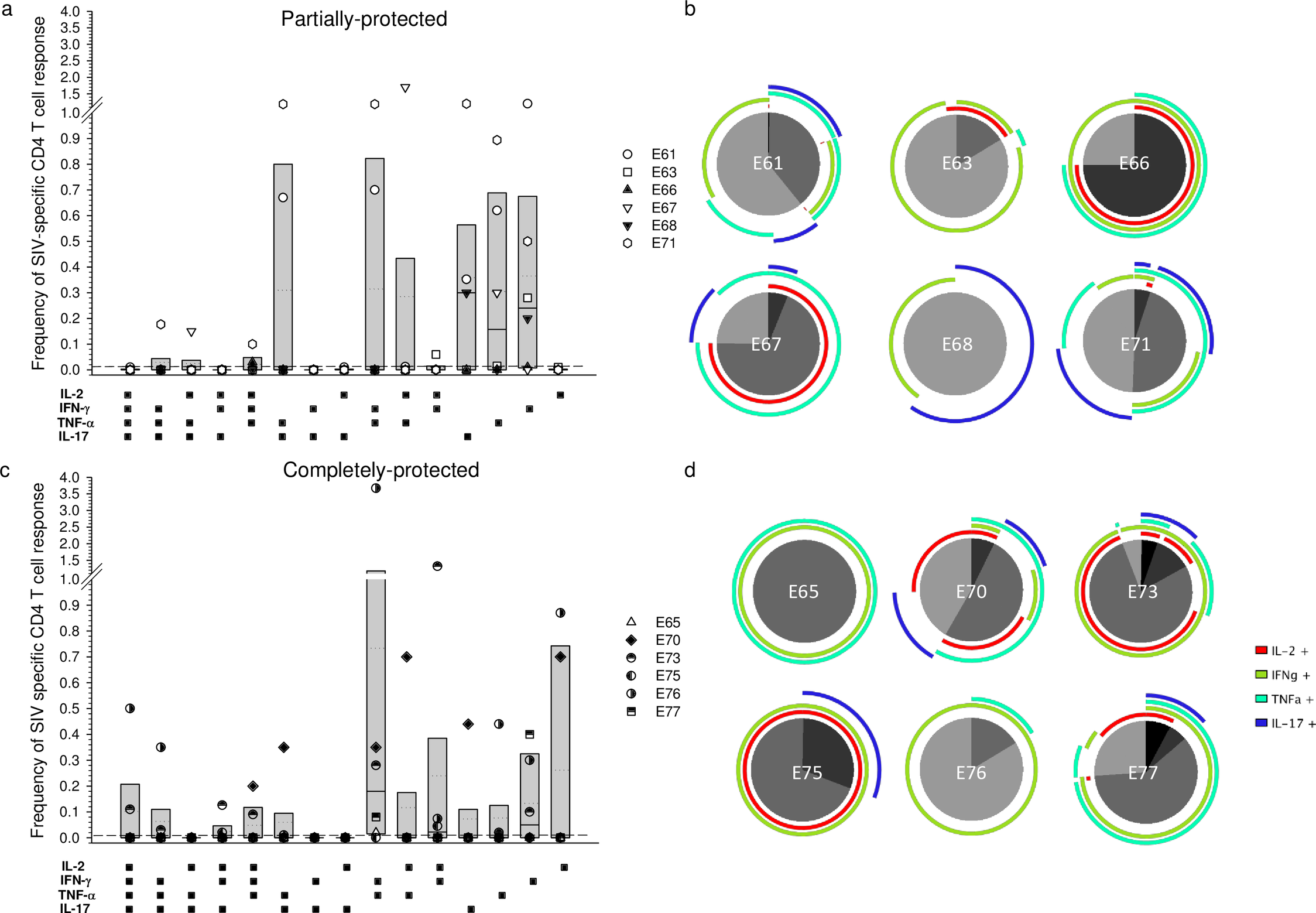

Supplement: S7 Fig — Cell populations were determined by multi-parametric flow cytometry following stimulation of PBMC separately with SIV-Gag, Rev and Tat peptide pools. Background responses detected in medium alone control samples were subtracted for every combination of cytokines and a cut-off of >0.01% after background subtraction was used as the threshold for positive reactivity (dashed line). Frequencies were derived by addition of results for Gag, Rev and Tat. Box plots show the 25th and 75th percentiles and median (solid line) and mean (dotted line) for each cytokine combination (a & c). Proportionate functionality for each macaque (b & d) is shown as a pie chart, with quadruple positivity shown in black and triple to mono positivity shown as shades of grey. Arcs show the combination of cytokine reactivities. (TIF) [file ppat.1006083.s007.tif]

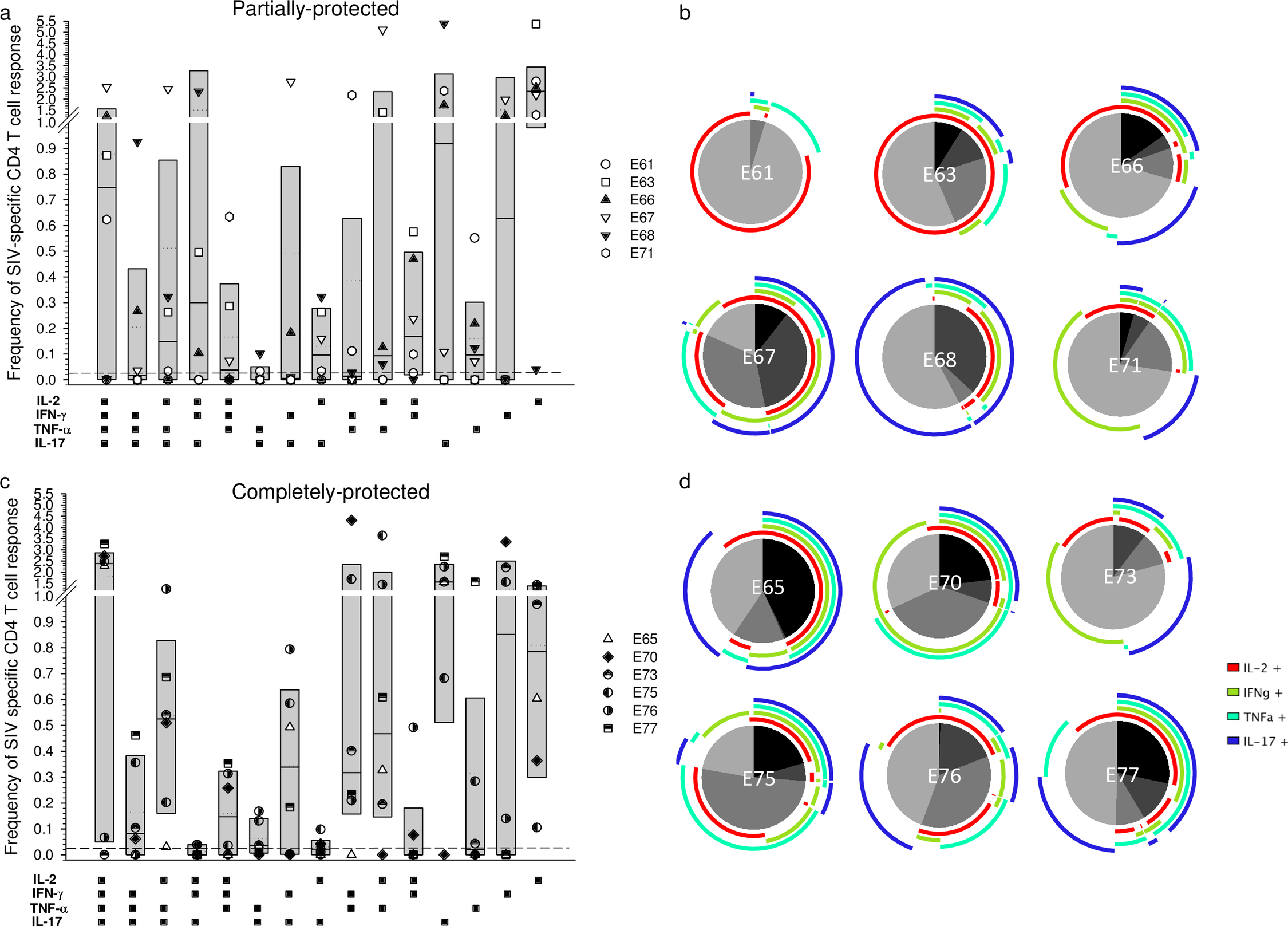

Supplement: S8 Fig — Cell populations were determined by multi-parametric flow cytometry following stimulation of PBMC separately with SIV-Gag, Rev and Tat peptide pools. Background responses detected in medium alone control samples were subtracted for every combination of cytokines and a cut-off of >0.01% after background subtraction was used as the threshold for positive reactivity (dashed line). Frequencies were derived by addition of results for Gag, Rev and Tat. Box plots show the 25th and 75th percentiles and median (solid line) and mean (dotted line) for each cytokine combination (a & c). Proportionate functionality for each macaque (b & d) is shown as a pie chart, with quadruple positivity shown in black and triple to mono positivity shown as shades of grey. Arcs show the combination of cytokine reactivities. (TIF) [file ppat.1006083.s008.tif]

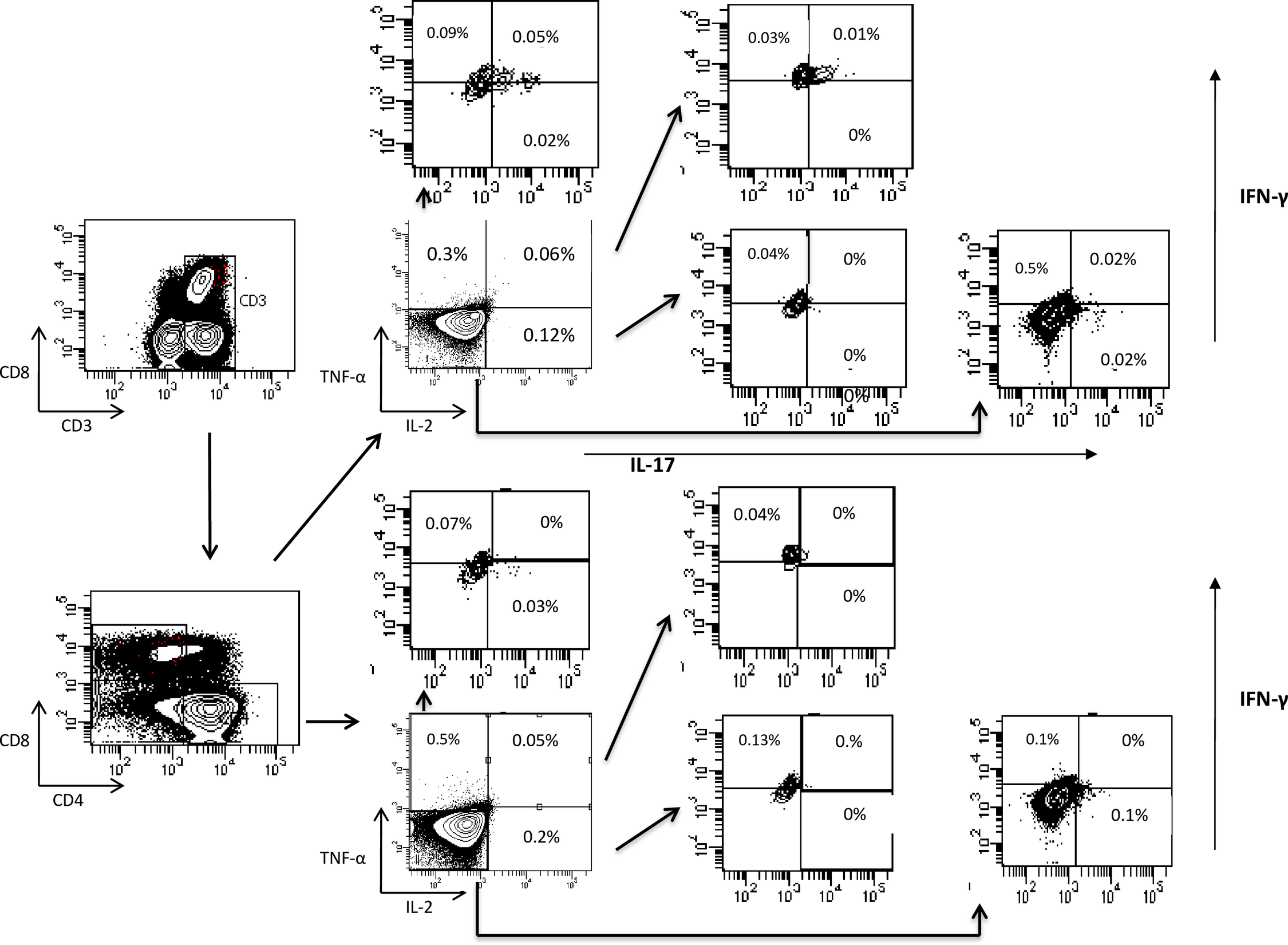

Supplement: S9 Fig — CD4 and CD8 subsets were defined via CD3. Within these subsets the distribution of TNF-α and/or IL-2 producing cells were specified using contour FACs profile quadrants. Each quadrant within these cell populations were sequentially analysed for IFN-γ and/or IL-17 production in combinatory plots. (TIF) [file ppat.1006083.s009.tif]
