# Supplementary material for: Role of Occult and Post-acute Phase Replication in Protective Immunity Induced with a Novel Live Attenuated SIV Vaccine
Source: PLoS Pathog. 2016 Dec 21;12(12):e1006083. doi: 10.1371/journal.ppat.1006083 (PMC5176322; doi:10.1371/journal.ppat.1006083)
Supplement: S1 Table — SIVrtTA RNA was recovered from plasma at several times after vaccination. The rtTA region was analyzed by RT-PCR and direct sequencing of the PCR product (population sequence). The LTR region was analyzed by RT-PCR, TA-cloning of the PCR product and sequencing of 9 to 12 TA clones. Silent and non-silent codon changes observed in the rtTA coding region are shown. The frequency at which mutations are observed in the LTR region is indicated between brackets (n.a., not analyzed). (DOCX) [file ppat.1006083.s010.docx]

| animal | week | rtTA | | | | LTR |
| --- | --- | --- | --- | --- | --- | --- |
| E61 | 2 |  |  |  |  | ∆NFkB-tetO (6/12) |
| E63 | 6 |  |  |  |  | n.a. |
|  | 12 |  |  |  |  | n.a. |
| E65 | 2 |  |  |  |  | n.a. |
|  | 6 |  | R80W (cgg-tgg) |  | E191K (gaa-aaa) | n.a. |
|  | 14 |  | R80W (cgg-tgg) | Y86Y (tac-tat) | E191K (gaa-aaa) | n.a. |
|  | 16 |  | R80W (cgg-tgg) |  | E191K (gaa-aaa) | ∆NFkB-tetO (10/10) |
| E66 | 2 |  |  |  |  | n.a. |
|  | 8 | n.a. | | | |  |
|  | 16 |  |  |  |  | n.a. |
| E67 | 2 |  |  |  |  |  |
| E68 | 2 |  |  |  |  | ∆NFkB-tetO (8/10) |
|  | 4 |  |  |  |  | n.a. |
| E70 | 2 |  |  |  |  | n.a. |
|  | 6 |  | R80Q (cgg-cag) |  |  | n.a. |
|  | 12 |  | R80W (cgg-tgg) |  |  | n.a. |
|  | 29 | L79L (ctg-cta) | R80W (cgg-tgg) |  |  | n.a. |
|  | 41 | L79L (ctg-cta) | R80W (cgg-tgg) |  |  | ∆NFkB-tetO (9/9) |
| E71 | 2 |  |  |  |  | n.a. |

**Table S1. Mutations in SIVrtTA observed upon *in vivo* replication.**
