# Supplementary material for: Role of Occult and Post-acute Phase Replication in Protective Immunity Induced with a Novel Live Attenuated SIV Vaccine
Source: PLoS Pathog. 2016 Dec 21;12(12):e1006083. doi: 10.1371/journal.ppat.1006083 (PMC5176322; doi:10.1371/journal.ppat.1006083)
Supplement: S2 Table — TRIM5/cyp status for each macaque was determined as indicated. X indicates inability to type this macaque. (DOCX) [file ppat.1006083.s011.docx]

|  | **MAMU MHC PROFILE FOR 9 ALLELES** | | | | | | | | | | **TRIM5 MAMU genotype** |
| --- | --- | --- | --- | --- | --- | --- | --- | --- | --- | --- | --- |
|  |  | **Class A** | | | | **Class B** | | | | |  |
|  |  | **A01** | **A02** | **A08** | **A11** | **B01** | **B03** | **B04** | **B08** | **B17** | **TRIM5 alleles** |
| **E61** |  | **+** |  |  |  |  |  |  |  |  | **mamu 4/ mamu 7** |
| **E62** |  |  | **+** |  |  | **+** |  |  |  |  | **mamu3/mamu7** |
| **E63** |  | **+** |  |  |  | **+** |  |  |  |  | **mamu 3** |
| **E64** |  |  |  |  | **+** | **+** |  |  |  |  | **mamu4/mamu7** |
| **E65** |  |  |  |  |  | **+** |  |  |  |  | **mamu4/mamu7** |
| **E66** |  |  | **+** |  |  | **+** |  |  |  |  | **mamu 4** |
| **E67** |  | **+** |  |  |  | **+** |  |  |  |  | **mamu4/ mamu X** |
| **E68** |  |  |  |  | **+** | **+** |  |  |  |  | **mamu 4/ mamu 7** |
| **E69** |  |  |  |  | **+** | **+** |  |  |  |  | **mamu3** |
| **E70** |  |  |  |  |  | **+** |  |  |  |  | **mamu 3/ mamu 7** |
| **E71** |  |  |  |  |  | **+** |  |  |  |  | **mamu 3/ mamu 4** |
| **E72** |  |  |  |  |  | **+** |  |  |  |  | **mamu 3/ mamu 7** |
| **E73** |  |  |  |  |  | **+** |  |  |  |  | **mamu 3/ mamu 4** |
| **E74** |  |  |  |  |  | **+** |  |  |  |  | **mamu 1/ mamu 4** |
| **E75** |  |  |  |  | **+** | **+** |  |  |  |  | **mamu 1/ mamu 4** |
| **E76** |  |  |  |  |  | **+** |  |  |  |  | **mamu 5** |
| **E77** |  | **+** |  |  |  | **+** |  |  |  |  | **mamu 1/ mamu 7** |
| **E78** |  | **+** |  |  |  | **+** |  |  |  |  | **mamu 3/ mamu 4** |
| **E79** |  |  |  |  |  | **+** |  |  |  |  | **mamu 5** |
| **E80** |  |  | **+** |  |  | **+** |  |  |  |  | **mamu 3/mamu 5** |
| **E81** |  | **+** |  |  |  | **+** |  |  |  |  | **mamu 3/mamu 5** |
| **E82** |  |  |  | **+** |  | **+** |  |  |  |  | **mamu 4** |

**Table S2**. **Mamu MHC profiles for 4 class A and 5 class B MHC alleles for all study vaccinates and challenge controls**
